# Recent insights into the morphology, molecular characterization and tissue localization of the caprine *Sarcocystis* species infecting domestic goats (*Capra hiricus*): *Sarcocystis moulei*, *Sarcocystis capracanis*, and *Sarcocystis hircicanis*

**DOI:** 10.1007/s00436-023-08063-3

**Published:** 2023-12-16

**Authors:** Ahmed El-Morsey, Walied Abdo

**Affiliations:** 1 Parasitology and Animal Diseases Department, Veterinary Research Institute, National Research Centre, 33 El Buhouth St. (former El-Tahrir St.), Dokki, Giza, P.O. 12622 Egypt; 2 Department of Pathology and Clinical Pathology, Faculty of Veterinary Medicine, Kafrelsheikh University, Kafr El-Sheikh, 33516 Egypt

**Keywords:** *Sarcocystis moulei*, *Sarcocystis capracanis*, *Sarcocystis hircicanis*, Ultrastructure, *Cox1*, Phylogeny

## Abstract

Ninety-seven (64.67%) out of 150 domestic goats (*Capra hiricus*) carcasses were found to be infected by *Sarcocystis moulei, Sarcocystis capracanis,* and *Sarcocystis hircicanis* sarcocysts. *Sarcocystis moulei* macrosarcocysts were detected in the cardiac, esophageal, skeletal, lingual, and diaphragmatic muscles of seven goats (4.67%) out of the 150 examined animals, whereas the microscopic *Sarcocystis* species were found in (90/150 = 60%). Two morphotypes of *S. moulei* were observed. Morphotype (I) macrosarcocysts were large-sized oval, ovoid, spherical, and measured 2–7 mm in length x 2–6 mm in width. *Sarcocystis moulei* morphotype (II) macrosarcocysts were spindle-shaped, spheroid, sometimes elongated, and measured 1.8–6 x 0.5–2 mm. By TEM, all *S. moulei* morphotypes were ultrastructurally the same and had a sarcocyst wall that was characterized by highly branched or cauliflower-like villar protrusions (VP) with dumbbell-like structures. The VP interior was packed with well-developed microtubules in longitudinal and cross arrangements. *Sarcocystis moulei* cyst wall was 3–6 μm thick. *Sarcocystis capracanis* microsarcocysts detected herein had a cyst wall that ranged from 4–8 μm in thickness. The VP was upright finger-like or cylindrical. The PVM had electron-dense corrugations in the region of the VP. Few amounts of microfilaments were detected inside the cores of VP. *Sarcocystis hircicanis* had a thinner cyst wall (~1–3 μm) with hairy long VP that ranged from 1 to 7.5 μm in length. Microtubules were missing inside the cores of the VP. The three caprine *Sarcocystis* species were molecularly characterized on the level of the *18S rRNA, 28S rRNA,* and *Cox1* genes.

**Supplementary Information:**

The online version contains supplementary material available at 10.1007/s00436-023-08063-3.

## Introduction

Species of the *Sarcocystis* have been gotten more attention within the previous 20 years as a result of recent discoveries of many novel species parasitizing both mammalian and avian intermediate hosts, high economic losses resulting from the condemnation of meat due to the existence of macroscopic cysts of some *Sarcocystis* species in several muscular organs. Macroscopic lesions associated with eosinophilic myositis as a consequence of bovine or ovine *Sarcocystis* spp. encystation in cattle, sheep, and goat musculature, or even merogony stages associated with granulomatous reactions might appear grossly as yellowish-white spots or cyst-like lesions, in some cases on the external surfaces of the internal organs such as the liver, kidney, lung, or spleen of the animal during carcass inspection in abattoirs (El-Morsey 2010; El-Seify et al. 2014; Dubey et al. 2016; Gjerde et al. 2020; El-Morsey et al. 2021). Additionally, the adverse pathogenic effects on the intermediate host as, abortion, fever, anemia, anorexia, and even deaths, more specifically due to infection by some of the canine-transmitted *Sarcocystis* spp. Up till now, more than 200 *Sarcocystis* spp. have been described in domesticated and wild animals. Some *Sarcocystis* species have considerable veterinary, economic, and public health importance (Dubey et al. 2015, 2016; El-Morsey 2010, 2016; El-Morsey et al. 2014; El-Morsey et al. 2015a, 2015b; 2019; 2021; Marandykina-Prakienė et al. 2022).

Domestic goats (*Capra hiricus*) act as intermediate hosts for three *Sarcocystis* spp., i.e., *S. capracanis, S. hircicanis,* and *S. moulei*. Both *S. capracanis* and *S. hircicanis* are characterized by the formation of microscopic sarcocysts that are transmitted by canines, whereas, *S. moulei* produces macrosarcocysts and is transmitted by felines (Dubey et al. 2016; El-Morsey et al. 2019).

The present study was performed with the objectives (i) to estimate the prevalence of sarcocystosis in domestic goats (*Capra hiricus*) slaughtered in Egypt; (ii) to investigate the tissue localization specificity of caprine *Sarcocystis* spp. in various muscular organs; and (iii) to perform a comprehensive morphologic and molecular identification of *S. moulei, S. capracanis,* and *S. hircicanis.*

## Materials and methods

### Animals and study region

One hundred and fifty domestic goats (*Capra hiricus*) (> 3 years old) carcasses were examined for the existence of macroscopic and microscopic caprine *Sarcocystis* species. The investigation was performed during the period extending from June 2021 till the end of January 2023. The ages of the inspected goat carcasses were determined through teeth examination. Animals were slaughtered and examined at El-Mahalla El-Kobra abattoir, Gharbia governorate, Egypt, (30° 58′ 07″ N 31° 09′ 49″ E). One hundred and eleven male and 39 female carcasses were inspected for the presence of both macroscopic and microscopic sarcocysts.

Different muscular organs including the tongue, esophagus, heart, diaphragm, and skeletal muscles were screened for the presence of *Sarcocystis* spp. macrosarcocysts by the naked eye under a strong source of light. Dimensions of the detected *Sarcocystis* species macrosarcocysts were measured using a transparent plastic ruler.

### Muscle squash method

For surveying the microsarcocyst forming *Sarcocystis* species, small fresh meat pieces selected from the formerly mentioned different muscular organs measuring 1–2 mm were compressed between two microscope slides. Slides were examined under the light microscope, (*OLYMPUS CX33®*), searching and performing preliminary morphologic diagnosis for the caprine microscopic *Sarcocystis* spp. under investigation.

### Impression smears from the macroscopic sarcocysts

The lingual, esophageal, skeletal, diaphragmatic, and cardiac muscles macrosarcocysts were carefully excised from the surrounding tissues and cut sharply in a cross-section using a sterile sharp scalpel blade. When the macrosarcocysts are pressed on a glass slide, an off-white or whitish fluid oozes. Such cyst fluid was spread on the microscope slide in addition the cut surface of the cysts was pressed on the slide, air dried, and fixed by absolute methanol then stained by Giemsa stain. Finally, stained cyst contents were examined by light microscope (*OLYMPUS CX33® Microscope Digital Unit*).

### Histopathological and ultrastructural morphologic characterization

#### Characterization of the detected morphotypes of *S. moulei* macrosarcocysts

Two macrosarcocysts belonging to each morphotype of *S. moulei* were isolated from the tongue, esophagus, heart, diaphragm, and skeletal muscles of each of the infected seven goat carcasses, i.e., four macrosarcocysts belonging to the two morphotypes were isolated from each muscular organ of the five. Hence, a total of 140 *S. moulei* macrosarcocysts, (70 for each morphotype), were isolated from the seven goats. Each macrosarcocyst of *S. moulei* was divided into two halves, using a sterile scalpel blade, on a clean sterile glass slide under the stereomicroscope. The first half was directly fixed in a small flask of formalin 10% while the second one was fixed in another flask of glutaraldehyde 2.5%. During the isolation and fixation of the cysts, both flasks were surrounded by ice within two larger jars. The fixed sarcocysts were kept at 4°C for subsequent histopathological and TEM examination.

#### Histopathologic examination

For histopathologic identification, the isolated *S. moulei* macrosarcocysts were fixed in 10% formalin, routinely processed, paraffin-embedded, cut into 5 μm sections, and stained with H&E. Micrographs were captured using *OLYMPUS CX33 Microscope Digital Unit.*

#### Ultrastructural examination

The isolated macrosarcocysts were processed for TEM as previously described by (El-Morsey 2010, 2016; El-Morsey et al. 2014, 2015a, 2019, 2021). Briefly, portions of the sarcocysts fixed in 2.5% glutaraldehyde were post-fixed in osmium tetroxide 1%, dehydrated in serially graded alcohols, and finally embedded in an epon-araldite mixture. Ultrathin sections were stained with uranyl acetate and lead citrate and then examined by *JEOL JEM-1400 TEM* at 80 kV.

#### Morphologic characterization of the detected *S. capracanis* and *S. hircicanis* microsarcocysts

Four mature microsarcocysts of both *S. capracanis* and *S. hircicanis* were excised carefully from the surrounding muscle tissues using a sterile needle under the light microscope. The microsarcocysts with striated thick walls having finger-like or cylindrical projections were assigned as *S. capracanis*, whereas cysts with thin smooth walls were assigned as *S. hircicanis*. The microsarcocysts were isolated from fresh muscle samples from the tongue, esophagus, heart, diaphragm, and skeletal muscles from a total of five different goat carcasses. Hence, a total of 100 cysts were selected from each species and directly processed in the same manner that was applied for *S. moulei* sarcocysts for histopathologic and ultrastructural identification. With the exclusion that, each microsarcocyst belonging to the two *Sarcocystis* spp. was entirely fixed (undivided cyst). The detected microsarcocysts of both *Sarcocystis* spp. were isolated from muscle samples collected from goat carcasses slaughtered for 6 months within the entire period of the prevalence study (1.5 years).

#### Molecular and phylogenetic characterization

The macrosarcocysts assigned as *S. moulei* were divided into two groups. Each group included one morphotype. A total of 104 macrosarcocysts were isolated from the seven *S. moulei*-positive goat carcasses. Fifty-four sarcocysts belonging to the two morphotypes, (27 sarcocysts for each morphotype), were isolated from the esophageal, lingual, cardiac, diaphragmatic, and skeletal muscles of the seven goat carcasses. The fifty-four macrosarcocysts were implemented for molecular characterization on the level of the *Cox1* gene by PCR. Similarly, the remaining 50 macrocysts were divided into two groups; the first one comprised 26 cysts while the second one contained 24 cysts. Group I and II were characterized on the levels of *18S rRNA* and *28S rRNA* genes, respectively.

Fifty *S. capracanis* microcysts were divided into three groups; group (I) comprised ten cysts to be tested by PCR on the level of *18S rRNA* gene, group (II) which was composed of 20 cysts for characterization on the level of *28S rRNA* gene and eventually group III that had 20 cysts to be tested on the level of *Cox1* gene. The 50 microcysts were isolated from five different goat carcasses, i.e., two separate microcysts from each of the lingual, esophageal, cardiac, diaphragmatic, and skeletal muscles of each carcass. In the same way, 47 microcysts assigned as *S. hircicanis* were divided into three groups. Groups I and II had 15 cysts each, whereas, group III included 17 cysts. The three sets were characterized on the levels of *18S rRNA, 28S rRNA,* and *Cox1* genes, respectively. All the isolated sarcocysts were preserved in 2-ml eppendorf® tubes containing ethanol 70% and kept in a deep freezer at -20° C until used for the molecular characterization.

#### DNA extraction, PCR, sequencing, and sequence analyses

Prior to DNA extraction, sarcocysts belonging to the three species were washed three times using sterile saline 0.9%. Genomic DNA was extracted using QIAGEN DNeasy Tissue Kit® (QIAGEN®, GmbH, Hilden, Germany) according to the manufacturer’s recommendations.

All PCR amplifications for the three target genes were performed in a total 50 μL reaction volume comprising 10X Ex Taq PCR buffer 5 μL, *Takara Ex Taq* (DNA polymerase, Takara Bio Inc. Japan) (5 units/1 μL) 0.25 μL, dNTP Mixture (2.5 mM each) 4 μL, 1 μL from each of the primers (0.4 μ M each), DNA template 5 μL and finally sterile double distilled water up to 50 μL. A free template (control tube) containing sterile distilled water was included in each PCR reaction.

For amplification of the small ribosomal subunit gene (*18S rRNA)*, 26 macrosarcocysts (13 from each morphotype) belonging to *S. moulei*, ten microsarcocysts of *S. capracanis*, and 15 *S. hircicanis* microcysts were incorporated into PCR amplifications. Sequences of the *18S rRNA* gene were amplified using the primers S1 (F)/B (R) designed by Fischer and Odening (1998) and (Medlin et al. 1988). Cycling conditions for the *18S rRNA* gene of the three *Sarcocystis* spp. were as follows: first hot start at 95° C for 15 min followed by 45 cycles of denaturation at 95 °C for 45 s, annealing at 56° C for 45 s, extension at 72° C for 90 s, and a final extension at 72° C for 10 min.

The large ribosomal subunit gene (*28S rRNA* gene) (~ 3500 bp) of 24 *S. moulei* macrosarcocysts (12 of each morphotype), 20 *S. capracanis* microcysts, and 15 microsarcocysts of *S. hircicanis* was amplified using the primer pair sets: KL1/KL3, KL4/KL5b, and KL6a/KL2 designed by (Mugridge et al. 1999). Conditions required for the replication of the *28S rRNA* gene were as follows: primary hot start at 95 °C for 3 min followed by 30 cycles of denaturation at 94 °C for 45 s, annealing at 55° for 45 s, and extension at 72 °C for 1 min, followed by a final extension at 72 °C for 5 min.

The Mitochondrial Cytochrome C Oxidase Subunit I gene (*Cox1* gene) for the three *Sarcocystis* species was amplified with the forward primer SF1 (F) and the reverse SR9 (R) (Gjerde 2013, 2014b). The DNA extracts of 54 macrocysts of *S. moulei* (27 from each morphotype), 20 *S. capracanis* microsarcocysts, and 17 microcysts of *S. hircicanis* were used as DNA templates in PCR.

Reaction cycling conditions were as follows: primary hot start at 95 °C for 15 min followed by 45 cycles of denaturation at 94 °C for 45 s, annealing at 52 °C for 45 s, extension at 72 °C for 90 s, and a final extension at 72 °C for 10 min.

All PCRs were performed in *TaKaRa PCR Thermal Cycler Dice*®^.^ (*Takara., Japan*). PCR amplification products were analyzed on 1.5% agarose gel.

PCR products were purified from gel using Qia Quick Gel Extraction kit® (QIAGEN®) according to the manufacturer’s protocol. Concentrations of DNA amplicons were estimated using NanoDrop ND-1000 Spectrophotometer (NanoDrop® Technologies, USA). One hundred and four macrosarcocysts of *S. moulei* (52 from each morphotype), 50 microsarcocysts of *S. capracanis,* and 47 *S. hircicanis* microcysts were analyzed on the levels of the three genetic markers, i.e., *18S rRNA, 28S rRNA,* and *Cox1* genes.

All the obtained isolates of the three *Sarcocystis* spp. were sequenced and analyzed using ABI 3730XL Automatic DNA Sequencer (Applied Biosystems, Foster City, CA, USA). The obtained *18S rRNA, 28S rRNA,* and *Cox1* sequences were compared to those from the National Center for Biotechnology Information (NCBI) https://blast.ncbi.nlm.nih.gov/Blast.cgi. Before performing the phylogenetic analyses, sequences were slightly truncated from both ends so that they begin and end with the same nucleotides.

### Phylogeny

Sequences of *S. moulei*, *S*. *capracanis,* and *S*. *hircicanis* obtained herein, along with the former related *Sarcocystis* spp. sequences, were initially aligned utilizing *ClustalW*. The *18S rRNA* and *Cox1* sequences of various *Sarcocystis* spp. infecting ruminants were downloaded from the database of GenBank. Phylogenetic analyses were performed on nucleotide sequences of *18S rRNA* and *Cox1* using MEGA 11 software (Tamura et al. 2021).

The evolutionary history of the *18S rRNA* gene sequences of the caprine *Sarcocystis* spp. identified herein was inferred using the Minimum Evolution (ME) method (Rzhetsky and Nei 1992). The bootstrap consensus tree reconstructed from 1000 replicates, (Felsenstein 1985), was taken to represent the evolutionary relationships of the taxa under investigation (Felsenstein 1985). Branches of the cladogram corresponding to partitions reproduced in < 50% bootstrap replicates were collapsed. The percentage of replicate trees in which the associated taxa clustered together in the bootstrap test (1000 replicates) were shown next to the branches or on the branching points (i.e., nodes) of the clades within the main tree (Felsenstein 1985). The evolutionary distances were computed using the Maximum Composite Likelihood method (Tamura et al. 2004) and are in the units of the number of nucleotide substitutions per site. The (ME) tree was searched using the Close-Neighbor-Interchange (CNI) algorithm by (Nei and Kumar 2000) at a search level of 1. The Neighbor-Joining Algorithm (Saitou and Nei 1987) was used to generate the initial tree. The current cladiastic analysis involved 47 nucleotide sequences. Forty-six *18S rRNA* sequences represented a total of 24 ruminant *Sarcocystis* spp. All ambiguous positions were removed for each sequence pair (Pairwise Deletion Option). There were a total of 1770 positions in the final dataset. The (ME) tree was based on *Neospora caninum* (**U03069**) as outgroup.

The phylogenic relationships on the level of *Cox1* gene sequences of the caprine *Sarcocystis* spp. detected herein were inferred by the (ME) method. The percentage of replicate trees in which the analyzed taxa associated together in the bootstrap test (1000 replicates) were shown next to the bifurcation points of the (ME) tree. The evolutionary distances were computed using the Maximum Composite Likelihood method and were in the units of the number of base substitutions per site. The ME tree was searched using the Close-Neighbor-Interchange (CNI) algorithm at a search level of 1. The cladiastic analysis included 44 nucleotide sequences. The ME analysis comprised 42 *Cox1* sequences representing 28 *Sarcocystis* spp. infecting both domesticated and wild ruminant intermediate hosts. All ambiguous positions were deleted for each sequence pair (Pairwise Deletion Option). There were a total of 1030 positions in the final dataset. Cladiastic analysis was conducted in MEGA11. The ME tree was rooted on *Toxoplasma gondii* isolates (**HM771689** and **HM771690**) as outgroup.

#### Statistical analysis

Prevalence data were statistically analyzed by one-way ANOVA and Tukey multiple comparisons using the software Graph Pad Prism 5. Statistical significance was acceptable at the level of *p* < 0.05.

## Results

The caprine *Sarcocystis* spp. (i.e., *S. moulei, S. capracanis*, and *S. hircicanis*) identified herein were existing in 97 (64.67%) out of a total of 150 slaughtered goat carcasses. *S. moulei* macrosarcocysts were detected in seven goat carcasses (4.67%) out of the 150 examined animals, while both *S. capracanis* and *S. hircicanis* microcysts were found in 90 (60%) out of the 150 inspected goat carcasses. Goat carcasses harboring only *S. capracanis* cysts were 51 out of 150 (34%). *S. hircicanis* microsarcocysts were found in 28 of 150 (18.67%). Dual microscopic *Sarcocystis* spp. infection by the two species was (11/150 = 7.33%) (Tables 1 and 3).

**Table 1:** Total infection rates of the three caprine *Sarcocystis* spp., microscopic *Sarcocystis* spp. infections compared to those of the macroscopic species, and the infection rates in female carcasses against those in males. Rates were significant at *p* < 0.05

	Number of examined animals	Number of infected animals	Number of goats having macroscopic *Sarcocystis* spp. infections	Number of goats having microscopic *Sarcocystis* spp.
Total	150	97 (64.67%)	7 (4.67%)	90 (60%)
Male	111	67 (44.67%)	2 (1.33%)	65 (43.33%)
Female	39	30 (20%)	5 (3.33%)	25 (16.67%)

The macrosarcocysts of *S. moulei* were detected in a total of seven goat carcasses, i.e., (five female and two male carcasses). Cysts were observed in most of the examined muscular organs including the lingual, esophageal, cardiac, diaphragmatic, and skeletal muscles. The overall macrosarcocyst tissue localization ratios in the inspected goat carcasses ranged from 71.42 to 100 % depending on the type of organ infected. All of the seven esophagii and hearts had *S. moulei* macrosarcocysts, followed by the skeletal muscles 6/7 (85.71%), and finally the lingual and diaphragmatic muscles 5/7 (71.42%), Table 2.

**Table 2:** The muscular tissue localization ratios of *S. moulei* macrosarcocysts in five target organs of the seven infected goats. Distribution rates were significant at *p* < 0.05

Muscular organ	Tongue	Esophagus	Heart	Diaphragm	Skeletal muscles
*S. moulei* localization ratio	5/7 (71.42%)	7/7 (100%)	7/7 (100%)	5/7 (71.42%)	6/7 (85.71%)

The microsarcocysts of *S. capracanis* were the most prevalent species in the esophageal muscles (51150/; 34%) followed by cardiac muscles (45/150; 30%), skeletal muscles (39/150; 26%), diaphragmatic muscles (33/150; 22%), and eventually the lingual muscles (21/150; 14%). On the other hand, the esophagus and the skeletal muscles contained the highest ratio of *S. hircicanis* cysts (28/150; 18.67 %, 27/150; 18%), respectively. *S. hircicanis* sarcocysts localization ratio in the cardiac muscles was (25/150; 16.67%). Lower *S. hircicanis* incidence ratios of (19/150; 12.67%) and (18/150; 12%) were observed in the diaphragmatic and lingual muscles, respectively. The tissue localization ratios of mixed infections by both, *S. capracanis* and *S. hircicanis* microcysts, were higher in the lingual and the esophageal (11/150; 7.33%) than those of the cardiac (9/150; 6%), skeletal (8/150; 5.33%), and the diaphragmatic muscles (5/150; 3.33), respectively, Table 3. The overall prevalence, the incidence rate of each *Sarcocystis* species among the total rates of microsarcocyst infections, and the tissue distribution ratios of the two *Sarcocystis* spp. in different goat muscular organs are shown in Table 3.

**Table 3:** Localization ratios of *S. capracanis* and *S. hircicanis* in various muscular organs, mixed infection rates, each microscopic *Sarcocystis* sp. infection rates in relation to the total rates of both species, and overall prevalences. Rates were significant at *p* < 0.05

Organs infected	*Sarcocystis* spp.					
	*S. capracanis*		*S. hircicanis*		Mixed infections	
	No	%	No	%	No	%
Tongue	21	14	18	12	11	7.33
Esophagus	51	34	28	18.67	11	7.33
Diaphragm	33	22	19	12.67	5	3.33
Heart	45	30	25	16.67	9	6
Skeletal muscles	39	26	27	18	8	5.33
Each species incidence in relation to total incidence of the microscopic spp.	51	56.66	28	31.11	11	12.22
Overall prevalence	51	34	28	18.67	11	7.33

### *Sarcocystis moulei *macrosarcocysts gross morphology

The macrosarcocysts of *S. moulei* were observed in most of the examined muscular tissues of seven out of 150 domestic goats (*Capra hiricus*). Cysts were predominantly localized in the esophageal, cardiac, skeletal, and diaphragmatic in addition to lingual muscles.

**Fig. 1:**
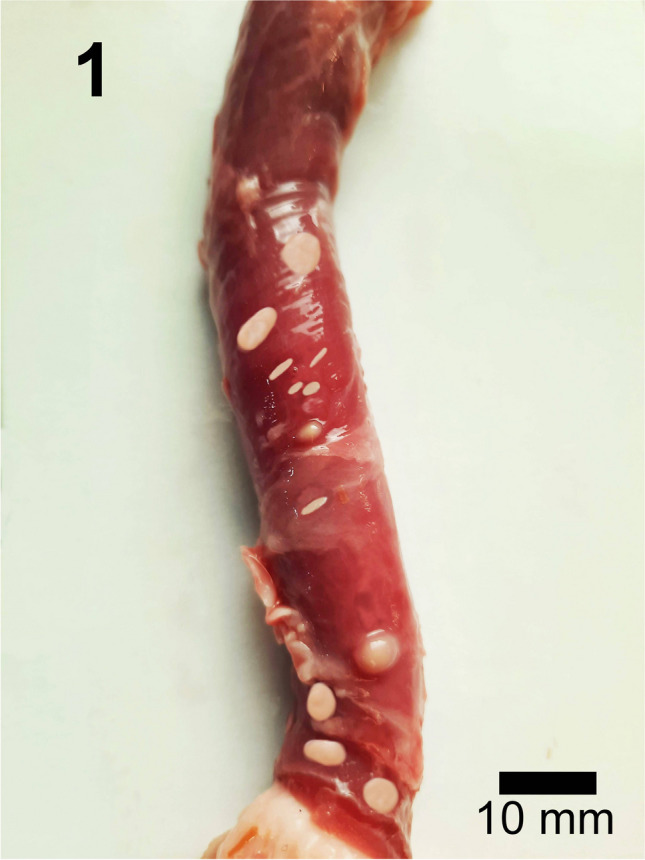
The macromorphology of *S. moulei* macrosarcocysts. A domestic goat (*Capra hiricus*) esophagus containing the two morphotypes of *S. moulei*. The macrocysts of morphotype (I) appeared larger in diameter and spherical, oval, ovoid, and yellowish-white in color. Morphotype (II) sarcocysts are smaller and elongated spindle or cylindrical in shape and whitish in color. Fresh muscular tissues. No stain. Scale bar = 10 mm

Two distinct morphotypes of *S. moulei* macrosarcocysts were observed in the current investigation. Morphotype (I) which was larger in diameter and ranged approximately from 2 to 7 mm in length x 2–6 mm in width (*n=50*). Macrosarcocysts had a mean length of 6.8 mm and a mean width of 3.2 mm. The macrosarcocysts of *S. moulei* belonging to morphotype (I) detected herein were oval, spherical, or ovoid-shaped cysts and were white, dull-white, and yellowish-white. Sarcocysts were mainly localized (from the highest to lowest in order) in the esophagus, cardiac muscles, skeletal, and to a little extent in the lingual and diaphragmatic muscles (Figs. 1, 1SF, 2SF, and 3SF). Morphotype (II) of *S. moulei* macrocysts was more spindle shaped, to little extent spheroid, mostly elongated, smaller in size, and measured approximately from 1.8–6 x 0.5–2 mm (*n=50*). Cysts had a mean length of 3.12 mm and a mean width of 1.2 mm. These macrosarcocysts were predominantly localized, (from the highest to lowest in order) in the cardiac, esophageal, lingual, skeletal, and to little extent in the diaphragmatic muscles (Figs. 1, 1SF, 2SF, and 3SF). Figures 1SF, 2SF, and 3SF with detailed gross morphologic descriptions of *S. moulei* macrosarcocysts in the cardiac, lingual, diaphragmatic, and skeletal muscles are included in the supplementary data file.

The macrosarcocysts of *S. moulei* were seen obviously in the cardiac muscles of the seven goat carcasses. *Sarcocystis moulei* cysts were found in all heart chambers including the walls of both auricles (Fig. 3SF). Macrosarcocysts were white or off-white. They were clearly seen under the endocardium of the right and left ventricles and deeply impeded in the myocardium of both ventricles and auricles as well (Figs. 3SFa, and 3SFb). In some cases, *S. moulei* macrosarcocysts were spheroid or broad spindle in shape and observed under the epicardium just on the outer surface of the heart muscles (Figs. 3SFc, and 3SFd). Sometimes, the cysts located under the endocardium appeared as spindle or fusiform in shape and white or dull white (Fig. 3SFb).

The macrosarcocysts of *S. moulei* were appearing as ovoid or elongated spindle-shaped off-white or white circumscribed cysts (morphotype II) immediately situated under the epicardium and within the myocardium of the heart (Figs. 3SFc, 3SFd).

*Sarcocystis moulei* macrosarcocysts were observed in the diaphragmatic muscles just under the serosal sheet of the diaphragm as oval and spherical white-colored cysts and ranged approximately from 3 to 5 mm in length (Morphotype I) (Fig.1SFc).

**Fig. 2:**
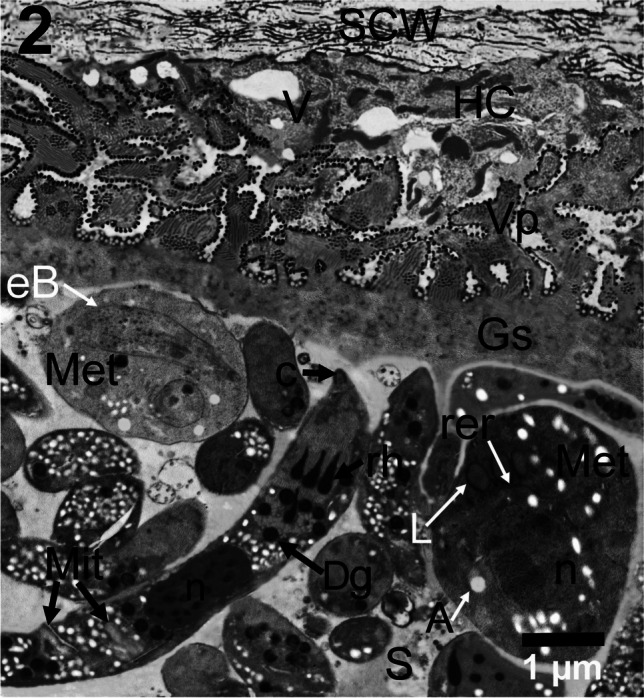
TEM micrograph depicting the ultrastructural characteristics of *S. moulei* macrosarcocysts detected in the current study. *Sarcocystis moulei* cyst wall is characterized by the existence of irregularly branched (VP) or sometimes cauliflower-like. Note the electron-dense irregular structures that are dispersed inside the (GS), vacuolation (V) in the myocytes due to degeneration of the infected host muscle cells (HC), secondary cyst wall composed mainly of dense collagen fibers (SCW), oval peripherally located electron-lucent metrocyte (Met) contains early developing bradyzoite (eB) during division by endodyogeny, electron lucent septa (S). Notice an elongated sickle-shaped bradyzoite containing conoid (c), 4 rhoptries (rh), dense granules (Dg), centrally located nucleus (n), and mitochondoria (Mit). Larger globular denser metrocyte (Met) containing lipid droplets (L), rough endoplasmic reticulum (rer), and nucleus (n) is also observed. Scale bar = 1 μm

In some cases, the esophageal and skeletal muscles harbored both morphotypes of *S. moulei* which ranged approximately from 1 to 5 mm in length and 1–3 mm in width. They were spindle-shaped, spherical, cylindrical, and white to yellowish-white. (Figs 1, 1SFd and Fig. 2SFa for Morphotype I) and (Fig. 2SFb for Morphotype II).

*Sarcocystis moulei* located in the lingual muscles were mainly seen on the ventral aspect of the tongue or deeply situated inside the core of the tongue. The macrosarcocysts were mainly spindle or cylindrical and yellow, yellowish-white, or white and measured ~1 – 3 mm in length (Morphotype II). In addition, a few ovoid or sub-spherical *S. moulei* morphotype (I) macrocysts were observed (Figs. 1SFa and 1SFb). Figures 1SF, 2SF, and 3SF with detailed gross morphologic descriptions of *S. moulei* macrosarcocysts in the cardiac, lingual, diaphragmatic, and skeletal muscles are incorporated in the supplementary data file.

### Impression smears from the detected macroscopic sarcocysts

The impression smears made from the detected cardiac and skeletal macrosarcocysts showed crescent, sickle-shaped, or banana-like deep blue or violet-stained bradyzoites. In addition, spherical, or ovoid sometimes irregularly shaped single or double nucleated (mostly metrocytes) were found. (Fig. 4SF).

### Muscle squash method

*Sarcocystis capracanis* cysts detected in the muscle squashes had cylindrical or long finger-like villar protrusions (VP). A compressed sarcocyst in an irregular form showing the cylindrically shaped VP on the outer surface of the cyst (Fig. 5SFa). A palisade-like villar protrusions were observed on the cyst wall that measured ~ 5–7 μm thick. A ribbon-like *S. capracanis* sarcocyst is depicted in Figure (5SFb).

The microsarcocysts of *S. hircicanis* had a thin cyst wall measuring approximately 1–3 μm without any obvious protrusions so that all the sarcocyst contents, i.e., bradyzoites (B) and metrocytes in addition to septae (S) were seen through the fine cyst wall (Fig. 5SFc). Figure 5SF with detailed morphologic descriptions is included in the supplementary data file.

### Histopathology

#### *Sarcocystis moulei*

*Sarcocystis moulei* macrosarcocysts had a thick fibrous connective tissue layer (FCT) that is immediately attached to the villar protrusions of the cyst wall. This layer is called the secondary cyst wall (SCW) and ranged from 5 to 10 μm in thickness. The FCT is formed mainly of fibrocytes and collagenous fibers. The primary cyst wall was 4–8 μm thick and had small irregular corrugated and branched villar protrusions (VP) (*n = 50*). Intact septa originated from the ground substance (Gs) of the cyst wall and divided the cyst into multiple chambers or compartments containing crescent or sickle-shaped long bradyzoites ranging from 12 to 16 μm (*n=40*). Many spaces were observed within the different cyst compartments. *Sarcocystis moulei* cystozoites were longer (12–16 μm) than those of *S. gigantea* that measured 5–9.5 μm. Moreover, the bradyzoites of *S. moulei* were more curved and nearly sickle-like or curved crescent-shaped (Fig. 6SFa).

#### *Sarcocystis capracanis*

Most of the detected microsarcocysts of *S. capracanis* were elongated ribbon shaped and few of them were spindle or spherical. Long sarcocysts, measuring from 100–800 μm long x 15–95 μm wide (*n* = 50), were found in most of the examined muscle samples. *S. capracanis* microcysts were characterized by a thick cyst wall that ranged from 4 to 7 μm (*n=35*). Elongated finger-like and cylindrical villar protrusions were characteristic of the sarcocysts of *S. capracanis* identified herein. The boundaries of the parasitophorous vacuolar membrane (PVM) in the region of the (VP) were deep blue stained, whereas the cores of the (VP) appeared white. A prominent eosinophilic ground substance layer (GS) was evident from which well-developed eosinophilic septa divided the cyst into many compartments containing bradyzoites. Most of the detected cysts of *S. capracanis* were mature that contained mainly banana-like zoites and few or sometimes no metrocytes (Fig. 6SFb).

#### *Sarcocystis hircicanis*

*Sarcocystis hircicanis* microscopic cysts had thin cyst walls that ranged from 1 to 2.5 μm in thickness. Hair-like VP appeared as originating from the ground substance GS of the cyst wall in most of the regions of the PVM, while a few sites appeared to have no VP. Due to the thin or weak architecture of the VP, they were wavier and get stuck to the surface of the sarcocyst, additionally, the GS appeared thicker than the total height of the villi. The VP was deep blue or violet stained and had no white or transparent cores when compared to those of *S. capracanis* as they were having lower breadth. The GS appeared eosinophilic and the septa as well. More spaces were also observed inside the interior of the cyst and banana-shaped zoites were also observed. Zoites of *S. hircicanis* ranged from 6 to 8 μm in length and were shorter than those of *S. moulei* (Fig. 6SFc). Figure 6SF can be found in the supplementary data file.

The initial physical examination done on goats before slaughter revealed that five of them were slightly emaciated and suffered from muscle weakness, stiffness of gait, and were reluctant to move. After slaughtering of these animals, their cardiac and skeletal muscles had some areas of color change, i.e., grayish-white foci were noticed. Light microscopic examination of muscle specimens comprising the grayish lesions showed high tissue intensity of *S. capracanis* microsarcocysts (more than four cysts in the field). Histopathological sections of the cardiac tissues from the five *S. capracanis*-positive goats showed the presence of variable degrees of cardiac muscle degeneration, necrosis, and fibrosis, particularly around the sarcocysts of *S. capracanis*. Mononuclear cell infiltrations mainly macrophages, and lymphocytes in addition to eosinophils were observed. Degenerating sarcocyst walls were also observed in some cysts after the migration of the inflammatory cells toward the cysts. Eventually, some degenerating sarcocysts and high degrees of muscle fibrosis were also observed (Fig. 7SF). Figure 7SF which depicts the caprine cardiac sarcocystosis micromorphology can be found in the supplementary data file.

Muscular infection by the microsarcocysts of *S. capracanis* might lead to the reduction of the meat quality and become unaesthetic for human consumption, because of degeneration, necrosis, mononuclear cell infiltrations (lymphocytes, macrophages), eosinophils around the microsarcocysts of *S. capracanis*, and finally fibrosis. Moreover, the aforementioned findings together with the clinical images described for the domestic goats examined before slaughter augmented the base that *S. capracanis* is a pathogenic species and might be able to induce a fatal cardiac sarcocystosis.

### TEM of the detected caprine *Sarcocystis *species

#### *Sarcocystis moulei*

The parasitophorous vacuolar membrane (PVM) of *S. moulei* was characterized by the formation of branched irregularly-shaped villar protrusions (VP). The (VP) together with the ground substance (GS) formed the primary sarcocyst wall (PCW). A fibrous connective tissue layer (FCT) that is consisted of electron-dense collagenous fibers was found firmly attached to the primary cyst wall. The (FCT) layer was named the secondary cyst wall (SCW) and ranged from 1 to 4 μm thick, sometimes a thickness of more than 4 μm was observed. A small layer of degenerated cardiac muscle fibers (host cells; HC) containing vesicles (V) and remnants of muscle fibers was observed in between the (PCW) and the (SCW). The (VP) appeared highly branched, mainly irregular in shape and sometimes cauliflower-like protrusions that contained well-developed several bundles of microtubules or fibrillar structures cut in both cross and longitudinal sections giving a tough supportive architecture to the (PCW). The (VP) height measured approximately 2–3 μm. The (PVM) in the region of protrusions is greatly undulated as a result of the existence of dumbbell-like deeply electron-dense structures (dbs) that are firmly attached only to the PVM. They are not out pocketing nor invaginations. The (PVM) located in between the VP had nearly two rows of spherical vesicular and globule-like structures that had electron lucent centers (VS). The GS thickness ranged from 0.5 to 1 μm. Electron dense particles (edp) that were variable in shape were found dispersed inside the GS layer. The total primary cyst wall thickness ranged from 3 to 6 μm (*n*=45). Electron lucent or lightly stained septa (S) of variable thickness originated from the GS and divided the cyst into compartments that contained elongated thin sickle-shaped bradyzoites (B). The bradyzoites contained many organelles comprising the conoid (c), rhoptries (rh), dense granules (Dg), amylopectin granules (A), centrally located nucleus (n), and mitochondoria (mit). Oval to spherical lightly stained metrocytes (Met) were observed. By TEM, the two morphotypes of *S. moulei* were ultrastructurally the same and had a sarcocyst wall that was characterized by highly branched or cauliflower-like VP with dumbbell-like structures (Fig. 2).

Metrocytes appeared as electron lucent oval or globular cells that measured 3–6 μm in length and 2–3 μm in width and contained spherical large-sized lightly stained nuclei (n), small-sized electron dense particles which could be ribosomes that are major components of the rough endoplasmic reticulum (rer), amylopectin granules (A) and lipid granules (L). An electron lucent nearly oval metrocyte (Met), that was just located under the (Gs) of the sarcocyst wall contained an early forming bradyzoite (eB), was observed (Fig. 2).

*Sarcocystis moulei* bradyzoites observed here were mostly sickle-like and ranged from 10 to 16 μm in length x 1 to 2 μm (*n*=60) in width with a centrally situated nucleus (n), several small-sized amylopectin granules (A) distributed in different sites inside the cytoplasm of the bradyzoite, mitochondoria (Mit), 4 rhoptries (rh), and an anterior cone-like conoid (C), many spherical electron-dense granules (Dg) located at both the anterior and posterior thirds and small sized thin micronemes (Mn) that were mainly localized in the anterior third of the bradyzoite (Fig. 9SF). Figures 8SF and 9SF with detailed ultrastructural features of *S. moulei* cyst wall and cystozoites are incorporated in the supplementary data file.

#### *Sarcocystis capracanis*

**Fig. 3:**
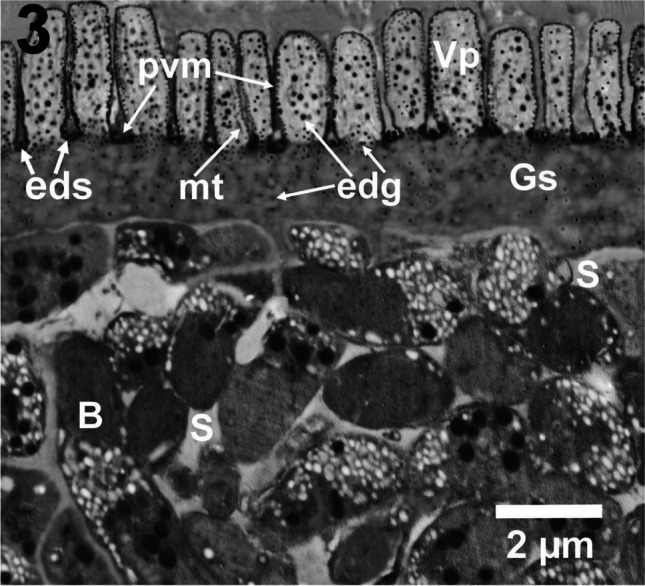
A photomicrograph depicting TEM of the microcysts of *S. capracanis* detected here. The sarcocyst wall of *S. capracanis* has finger-like or cylindrical (Vp) containing microfilaments (mt), electron-dense granules (edg) of varying sizes, electron-dense (pvm) that is characterized by corrugations on the (Vp). Note the electron-dense structures (eds) situated in between the (Vp), electron-dense granules scattered within the ground substance (GS) and more concentrated toward the bases of the villi, electron-lucent septa (S), and bradyzoites (B) in different sections directions within the cavity of the cyst. Scale bar = 2 μm

The sarcocyst wall of *S. capracanis* ranged from 3.65 to 4.95 μm in thickness. The VP of the cyst wall appeared upright finger-like or cylindrical. Variable distances in between the VP were evident and ranged from 50 to 500 nm. The PVM appeared as an electron-dense thin layer surrounding the outer surface of both the region of the VP and the sarcocyst surface in between the villi. Most of the PVM had many electron-dense corrugations in the region of the VP. Deeply stained or electron-dense oval or rounded structures (eds) were found in between the VP on the surface of the sarcocyst. The GS ranged from 1.3 to 2.2 μm in thickness. The GS contained electron-dense granules (edg), which were variable in their distribution as they were crowded toward the bases of the VP and few in other regions of the GS. The (edg) in the core of the VP were variable in size and included small and large-sized granules. Few amounts of microfilaments or microtubules (mt) were observed within the cores of VP. Electron-lucent septa (S) compartmentalized the sarcocyst into chambers containing banana-shaped bradyzoites (B) (Fig. 3).

*Sarcocystis capracanis* bradyzoites were banana-like and ranged from 8 to 11 μm in length x 2–2.5 μm in width. The bradyzoites contained anteriorly located conoid (c) that measured ~ 600 at its base and ~ 650 nm in length, micronemes (mn) located mainly at the anterior quarter, seven rhoptries (Rh), electron-dense granules (Dg) that were lower in number than those of *S. moulei* bradyzoites, Mitochondoria (Mit), amylopectin granules (A) those were mainly located in the middle third of *S. capracanis* bradyzoites, and posteriorly located nucleus (n) that was larger than that of *S. moulei* bradyzoites. *Sarcocystis capracanis* bradyzoite ultrastructure is included in the supplementary file under number 10SF.

#### *Sarcocystis hircicanis*

**Fig. 4:**
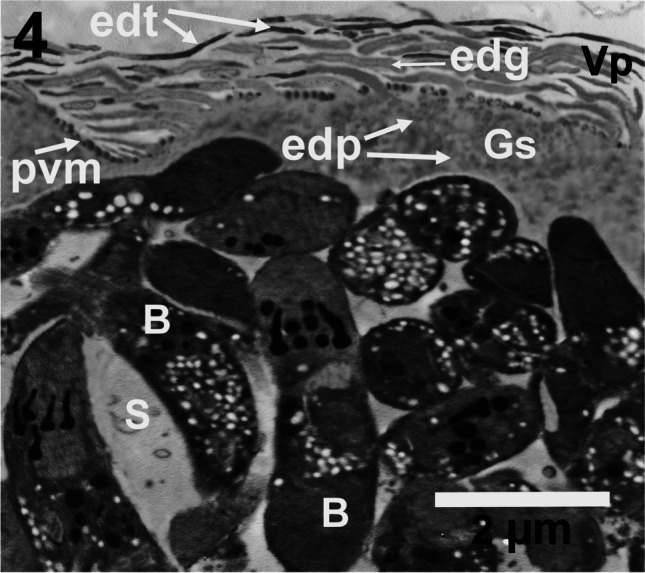
TEM micrograph showing *S. hircicanis* cyst wall with hair-like villar protrusions (Vp) that appears wavy and bent to the surface of the sarcocyst. The (Vp) cores contain only tiny electron-dense granules (edg) and have a broader origin or proximal third and very thin electron dense tips (edt) of the distal third. The (pvm) in the interspaces between the (Vp) has evident electron dense structures. Electron dense particles (edp) of varying sizes were dispersed within the ground substance (Gs). Note the electron lucent septa (S) and short bradyzoites (B) containing five drum stick-like rhoptries. Scale bar = 2 μm

Hairy VP is characteristic of the cyst wall of *S. hircicanis*. The VP could be divided into three portions. The first proximal third is wider than both the second and the third one that tapers distally for a long distance. The distal portions or the electron dense tips (edt) of the VP are electron dense or osmiophilic or deeply stained and appeared dense black. Very fine electron dense granules (edg) were found dispersed all over the cores of VP and might be more aggregated within the distal portions of VP. Such concentration or aggregation might be the cause of the black appearance of the distal thirds of the VP. The hairy long VP ranged from 1 to 7.5 μm in length. Microtubules were absent inside the cores of the VP resulting in a weak architecture of the VP, so they were usually bent and get adhered to the surface of the sarcocyst wall giving it a thinner appearance especially when compared to those of *S. moulei* and *S. capracanis*. Electron-dense projections were observed in the interspaces between the VP on the outer surface of the PVM. Prominent electron-dense particles (edp) of variable dimensions, ranging from (~ 100–200 nm), were dispersed within the GS. The total thickness of the cyst wall ranged from 1 to 3 μm. The cyst interior was compartmentalized by septa (S) into many chambers containing bradyzoites (B) (Fig. 4).

*Sarcocystis hircicanis* bradyzoite measured 6–8 μm in length x 1–2 μm in width and contained posteriorly located nucleus (n), amylopectin granules (A) those were concentrated mainly anterior to the nucleus, five rhoptries (Rh) those possess drum stick-like posterior ends, dense granules (Dg), anteriorly situated conoid (c) measuring approximately 100–150 nm at the anterior end and 300 nm at its base, and micronemes (mn) located mainly within the anterior fifth of the bradyzoite (Fig. 11SF). Figure 11SF which depicts the fine structure of *S. hircicanis* bradyzoite can be found in the supplementary data file.

### The caprine *Sarcocystis *spp. sequence analyses

#### *S. moulei 18S rRNA *sequences

An identity of 99.89% was observed between isolate OP430827 (sm55) and OP430830 (sm66) in contrast, a little bit lower similarity (99.78%) was observed with isolate (L76473) of *S. moulei* reported previously from goats in Germany by Jeffries et al. (1997). Intraspecific sequence identities of 99.62%, 99.46%, and 99.26% were observed between *S. moulei* isolate OP430827 (sm55) and isolates; OP430832 (sm77), OP430834 (sm88), and OP430835 (sm99) of the same species, respectively. Interspecific identities of 98.59% and 98.22% were observed between the currently identified *S. moulei* isolate OP430827 (sm55) and *S. gigantea 18S rRNA* sequences MK420020 (isolate OS13 from sheep in Spain; Gjerde et al. 2020), MT705975 (isolate AR6 from Egypt; El-Morsey et al. 2021), respectively (Table 5). Two nucleotide substitutions inform of transitions (Ts) were observed when the present isolate (OP430827; sm55) was aligned against (sm66). Four (Ts) were observed with *S. moulei* isolate L76473, followed by seven (Ts) with (sm77), 8 (Ts) with (sm88), and eventually 12 (Ts) with isolate (sm99) (Tables 4 and 5).

**Table 4:** Names, accession numbers, and the number of obtained isolates, for *S. moulei, S. capracanis,* and *S. hircicanis* on the level of *18S rRNA, 28S rRNA,* and *Cox1* genes

	*18S rRNA*	*28S rRNA*	*Cox1*
*S. moulei*	(sm55)-OP430827-(5) (sm66)-OP430830-(5) (sm77)-OP430832-(5) (sm88)-OP430834-(5) (sm99)-OP430835-(6)	SM (116)-OP429586-(6) SM (118)-OP430799-(3) SM (120)-OP430800-(5) SM (122)-OP430801-(3) SM (124)-OP430802-(4) SM (126)-OP430803-(3)	(SMn1)-OP485101-(29) (SMn2)-OP485102-(22) (SMn3)-OP485103-(1) (SMn4)-OP485104-(1) (SMn5)-OP485105-(1)
*S. capracanis*	(sc11)-OP430804-(1) (sc22)-OP430806-(1) (sc33)-OP430808-(5) (sc44)-OP430809-(3)	(sc100)-OP425732-(7) (sc102)-OP425798-(3) (sc104)-OP425811-(5) (sc106)-OP426272-(5)	(SC1)-OP470343-(13) (SC2)-OP470344-(7)
*S. hircicanis*	(sh11)-OP430816-(1) (sh22)-OP430818-(1) (sh33)-OP430820-(7) (sh44)-OP430823-(6)	(sh108)-OP426444-(8) (sh110)-OP429224-(3) (sh112)-OP429423-(2) (sh114)-OP429424-(2)	(SH1)-OP470341-(9) (SH2)-OP470342-(8)

#### *S. moulei 28S rRNA* sequences

Twenty-four *28S rRNA* sequences were obtained from 24 different macrocysts of *S. moulei*. The intraspecific variations ranged from 0.09 to 0.29 % among the 24 sequences. Such variations included two nucleotide transitions (Ts) when the isolate SM116 was compared with (SM118), three (Ts) with (SM120), five with (SM122), seven with (SM124), and eight with (SM126). *Sarcocystis moulei 28S rRNA* sequence (AF012884) detected in former investigation, (Mugridge et al. 1999; 2000), showed an identity percentage of 99.97 with the isolate (SM116; OP429586) identified in the current study. Sequence similarities of 99.91%, 99.89%, 99.86 %, 99.80%, and 99.71% were observed between isolates OP430799 (SM118), OP430800 (SM120), OP430801 (SM122), OP430802 (SM124), and OP430803 (SM126) of *S. moulei*, respectively when compared with isolate OP429586 (SM116) of the same species. On the other hand, an interspecific identity of 97.89% was observed between isolate OP429586 (SM116) and both isolates (AF044250) and (U85706) of *S. arieticanis* and *S. gigantea*. The isolate AF044250 was found to be the reverse sequence of *S. gigantea*. (Gjerde et al. 2020). Previously published sequences of *S. gigantea* under accessions MK420025 and MT706045 had sequence homologies of 97.87% and 97.52%, respectively as compared to the isolate (SM116) identified in the present investigation. *Sarcocystis medusiformis* isolate (MT706454; AR22) identified in domestic sheep (*Ovis aries*) from Egypt by El-Morsey et al. (2021) shared an interspecific identity of 94.31% with *S. moulei* isolate (SM116) detected herein (Tables 4 and 5).

#### *S. moulei Cox1 *sequences

Five haplotypes of *Cox1* sequences were obtained from a total of 54 different sarcocysts of *S. moulei*. Twenty-seven sequences were obtained from each of the two morphotypes of *S. moulei* (Tables 4 and 6).

Intraspecific identities ranging from 99.81 to 98.55% were observed when *S. moulei Cox1* isolate OP485101 (SMn1) was compared with the remaining isolates of the same species, i.e., OP485102 (SMn2), OP485103 (SMn3), OP485104 (SMn4), and OP485105 (SMn5). The intraspecific variations among the 54 *Cox1* isolates of *S. moulei* were in the form of two nucleotide transversions (Tv) between (SMn1) and (SMn2), while 6 transitions (Ts) and 4 (Tv) were observed when (SMn1) was aligned against (SMn3). Eight nucleotide transitions and five transversions were observed between (SMn1) and (SMn4). On the other hand, nine (Ts) and six (Tv) were found when (SMn1) was compared to (SMn5) (Tables 4 and 6). Nonetheless, such nucleotide variations were not reflected in the fine structural characteristics of the sarcocysts of the two morphotypes. Accordingly, all the identified macrosarcocysts belonged to a single species, i.e., *S. moulei*. An interspecific sequence identity of 91.91 % was observed when the isolate (SMn1) was compared to isolate MK420011 (OS7) of *S. gigantea* reported by Gjerde et al. (2020). Lower identities were found between the current isolate OP485101 (SMn1) and *S. gigantea* MT722969 (AR28) (91.62%) and MT722970 (AR30) (91.52%) (Table 6). Hence, the ovine macrocyst forming *S. gigantea* was the most similar *Sarcocystis* species to *S. moulei* isolates identified herein.

Detailed sequence identities concerning *S. moulei, S. capracanis*, and *S. hircicanis* isolates identified in the current investigation compared to the previously GenBank published sequences of the corresponding and most similar *Sarcocystis* species on the level of the three genetic markers *18S rRNA, 28S rRNA,* and *Cox1* genes are included in Tables 5 and 6 and can be found in the supplementary data file.

#### Cladiastic analyses

The isolates of *S. moulei, S. capracanis,* and *S. hircicanis* identified herein on the levels of *18S rRNA* and *Cox1* genes were grouped with the formerly detected isolates of the corresponding and the most related *Sarcocystis* spp. in the same clades. The five *S. moulei 18S rRNA* isolates (OP430827; sm55, OP430830; sm66, OP430832; sm77, OP430834; sm88 and OP430835; sm99) were clustered with the previously identified isolate (L76473) of the same species. The *18S rRNA* gene sequences of *S. capracanis* (OP430804; sc11, OP430806; sc22, OP430808; sc33, and OP430809; sc44) were placed as sister taxa to the previously recorded sequences (KU820982, KU820983, and MW832493) of *S. capracanis*. A robust association was formed between *S. hircicanis* isolates (KU820984 and KU820985) and the isolates of the respective species investigated here: OP4308016; sh11, OP4308018; sh22, OP430820; sh33 and OP430823; sh44) (Fig. 12SF). Figure 12SF regarding the *18S rRNA* sequences cladogram is incorporated in the supplementary data file.

**Fig. 5:**
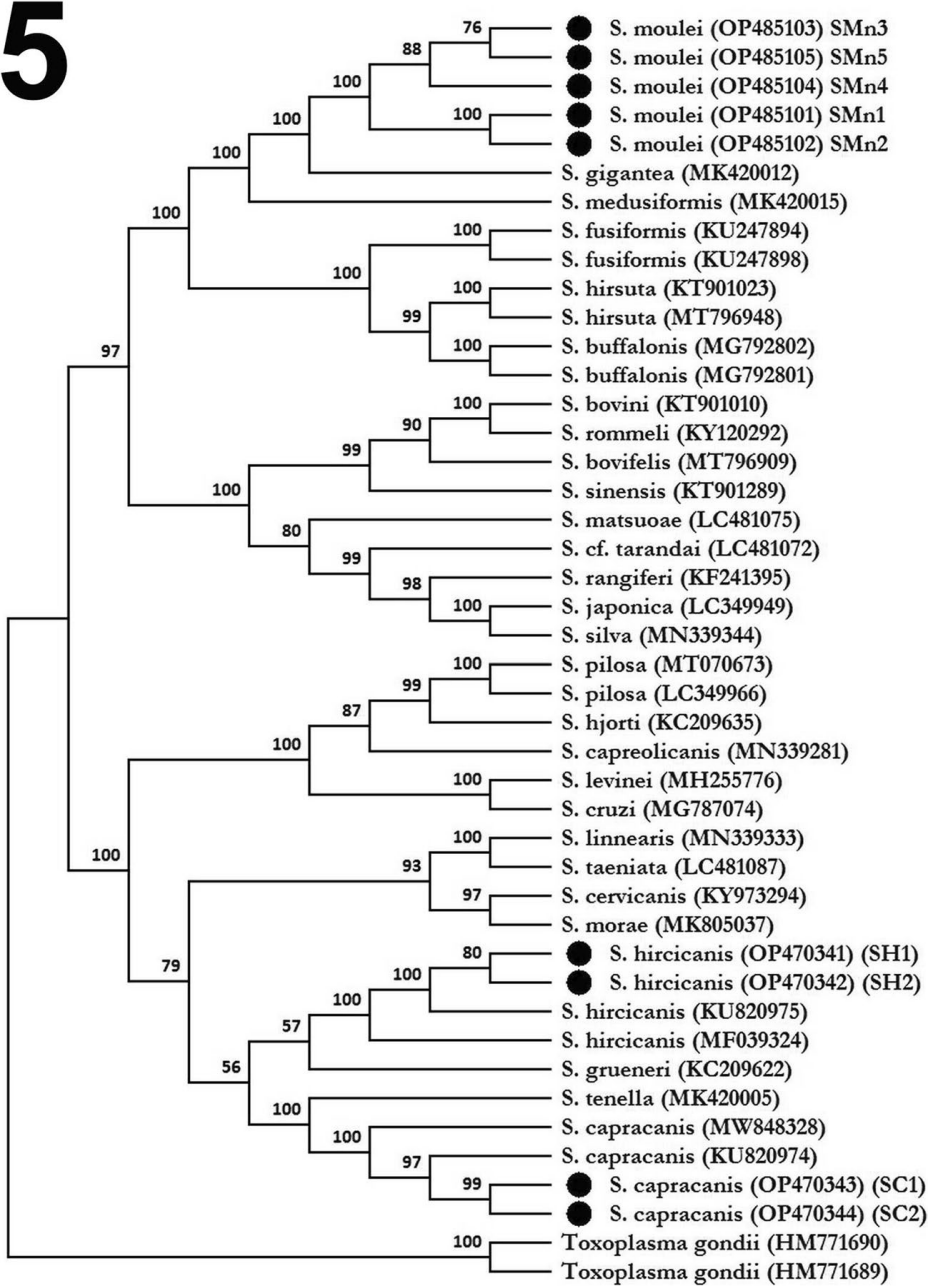
Phylogenic tree of the *Cox1* sequences of the currently identified isolates of *S. moulei, S. capracanis,* and *S. hircicanis*. The tree was based on *Toxoplasma gondii* isolates (HM771689 and HM771690) as outgroup. The isolates of the three caprine *Sarcocystis* species identified in the present investigation are marked by black circles just before the taxa names. In addition, haplotype names are written in parenthesis after the accession numbers

A well-supported clade was formed by the grouping of the *S. moulei Cox1* isolates identified herein (Fig. 5). Such clade was robustly associated together with an outer branch formed by *S. gigantea* isolate (MK420012) with a bootstrap support value of 100 reflecting the correlation between the two taxa on both the genetic and phenotypic characters (i.e., morphologic and structural levels) as the two species shared few similarities in the cyst wall morphology. Nonetheless, the grouping of the isolates of the two taxa relied on the cornerstone of sharing the same feline definitive hosts or cats. Similarly, *Cox1* isolates of *S. capracanis* and *S. hircicanis* identified herein were strongly associated in well-supported clades formed by the canine-transmitted isolates of the same species. The variant numbers of nucleotide transitions and/or transversions Ts/Tv among the analyzed *18S rRNA* and *Cox1* sequences did not affect the topologies of the phylogenic trees, i.e., the same *Sarcocystis* spp. localizations and the grouping or the associations of the currently identified isolates.

## Discussion

The three caprine *Sarcocystis* spp. identified herein were found in 97 (64.67%) out of a total of 150 slaughtered goat carcasses. The macrosarcocysts of *S. moulei* were detected in seven goat carcasses (4.67%) out of the 150 examined animals. Variable infection rates of the microscopic caprine *Sarcocystis* spp. were reported in previous investigations. *Sarcocystis capracanis* microcysts were observed by (Morsy et al. 2011) in 540 (79.4%) out of 680 examined goat carcasses slaughtered in Cairo, Egypt. *Sarcocystis capracanis* sarcocysts were found in 91.6% of 120 goat carcasses slaughtered in Brazil by Bittencourt et al. (2016). Hu et al. (2016) reported *S. capracanis* and *S. hircicanis* microcysts in 174 (77.3 %) out of 225 domestic goats from China.

*Sarcocystis moulei* macrosarcocysts were found in seven goat carcasses comprising five females (~7 years old) and two males (~5 years old). The infection rate in females in the current investigation was found to be higher than that of males this might be due to the productive life span is generally longer in female goats (~ 8–10 years) than that of males (~ 6–8 years). The long life span of the animal might give more chances for exposure to greater doses of the infective *Sarcocystis* spp. sporocysts in the contaminated pasture. In addition, the older the animal the longer the time needed for enough development and continuous growth of the sarcocysts to the macroscopic level as the macrosarcocysts might need more than 7 years to develop and grossly appear (Heydorn and Kirmse 1996; Dubey et al. 2016; El-Morsey et al. 2021).

*Sarcocystis moulei* fat macrocysts identified in Iraqi domestic goats by Barham et al. (2005) were exclusively found in the esophageal muscles. Nonetheless, *S. moulei* two morphotypes identified here were localized in the esophageal, cardiac, skeletal, lingual, and diaphragmatic muscles.

Most of the macrosarcocysts of *S. moulei* localized in the heart muscles were belonging to morphotype (II). Such macrocysts were mainly spindle and somewhat elongated in shape, this might be due to the continuous adaptation that occurs to the sarcocyst as a result of the higher continuous activity or contractility of the cardiac muscles when compared to other muscular organs. Additionally, the cardiac muscle fibers or the cardiomyocytes are strongly entangled with each other thus resulting in augmentation of longitudinal pressure on the early developing sarcocysts leading to more elongation. *Sarcocystis moulei* thin macrosarcocysts, detected by Barham et al. (2005), were missing in the cardiac muscles.

Both of the morphotypes of *S. moulei* macrosarcocysts identified herein were morphologically the same on the levels of histological and ultrastructural features. On the other hand, both, fat (large-sized) and thin (small-sized) macrocysts, reported previously by Barham et al. (2005) represented two different species relying only on the morphologic characters of the cyst bradyzoites by the impression smears. The size differences in bradyzoites were minor and overlapping. The findings in the present paper based on the comparative *Cox1* sequence analyses provided conclusive data that both species are the same.

The ultrastructural morphologic characters of the sarcocyst wall are the basic criteria used for the speciation and delineation of all *Sarcocystis* species infecting animals, birds, and reptiles. In addition, the genetic variations and the phylogenetic correlations among the sequences of *18S rRNA, 28S rRNA,* and *Cox1* genes are considered cornerstones for the comprehensive identification of the *Sarcocystis* spp. possessing some morphologic similarities, particularly the species infecting related intermediate hosts (Dubey et al. 1989; Dubey and Odening 2001; Dubey et al. 2015, 2016; Hilali et al. 2011; Prakas et al. 2016, 2017, 2019; El-Morsey 2010, 2016; El-Morsey et al. 2014; El-Seify et al. 2014; Gjerde 2014a, 2014b; El-Morsey et al. 2015a, 2015b; 2019; 2021).

The ultrastructural features of *S. moulei* macrosarcocysts described herein were somewhat similar to those reported by Ghaffar et al. (1989) in naturally and experimentally infected goats from Egypt, Afghanistan, Saudi Arabia, and Germany. Nonetheless, the sarcocyst wall detected herein was thinner. It ranged from 3 to 6 μm in thickness whereas, it was 7–8.5 μm in the sarcocysts reported by Ghaffar et al. (1989). The ground substance was thicker, (3–4 μm), than that was observed here (0.5–1 μm). The electron-dense particles in the (GS) of *S. moulei* identified here were fewer and irregularly shaped while it was numerous, spherical, and measured 500–1000 nm in diameter in *S. moulei* sarcocysts reported by Ghaffar et al. (1989). *S. moulei* identified herein had eminent dumbbell-like deeply electron-dense structures (dbs) that appeared firmly attached only to the PVM. They were not out pocketing nor invaginations. Such structures gave the PVM a well-developed and clear undulating appearance while those structures were not evident or completely absent in the PVM of *S. moulei* cysts previously described by Ghaffar et al. (1989). Additionally, the (PVM) located in between the VP of the currently identified *S. moulei* cysts, had nearly two rows of spherical or vesicular and globule-like structures that had electron lucent centers (VS). Those structures were not existing on *S. moulei* PVM detected by Ghaffar et al. (1989), in which, the PVM had only alternative thick and thin sites instead. *S. moulei* macrosarcocysts identified in the present investigation had cyst wall characteristics that are concordant to some degree with cyst wall type-21 according to (Dubey et al. 2016).

As comparing the current findings regarding the caprine *Sarcocystis* spp. tissue localization diverse ratios were recorded in previous investigations. Barham et al. (2005) detected *S. moulei* macrocysts in 33.6% of domestic goats in Iraq which were mainly found in the esophagus. It contained macrocysts in 98.9% (275/278) of all infected goats while the skeletal muscle contained macrocysts in 4.3% (12/278) and diaphragm with macrocysts in 2.5% (7/278) of the screened goat carcasses. Shekarforoush et al. (2005) detected *S. moulei* in 28 out of 169 goats (16.6%) in Iran. The macrocysts were mainly found in the esophagus, diaphragm, and tongue while the examined hearts were free. Dafedar et al. (2008) reported that the muscular organ localization ratios of both *S. capracanis* and *S. hircicanis* were 69.45%, 36.10%, 62.50%, and 12.5 % in esophagus, heart, diaphragm, and tongue, respectively. Moreover, Morsy et al. (2011) found that *S. capracanis* highest infection rate was recorded in the skeletal muscles (77%) followed by the diaphragm (74%), esophagus (54.4%), tongue (43.5%) while the lower rate was recorded in heart muscles (38%).

Discrepancies in *Sarcocystis* spp. muscular organ distribution might be due to oocyst contamination, varieties of *Sarcocystis* spp. isolates responsible for the infection, variations in the host tissue affinities for different *Sarcocystis* spp. and their isolates, and differences in the ecological and nutritional status of the hosts may lead to variations in the immune response against the infection and parasites as well (Shazly 2000; Daugschies et al. 2000; Mehlhorn 2008; Abdel-Ghaffar et al. 2009).

Comparative analyses of the currently obtained sequences of the *18S rRNA, 28S rRNA,* and *Cox1* genes for the three caprine *Sarcocystis* species have confirmed the relatedness of these isolates to the previously published isolates of the corresponding or other ruminant *Sarcocystis* species on GenBank. Additionally, the *Cox1* sequences detected herein for *S. moulei* macrosarcocysts are considered the premier global record on GenBank. Furthermore, our findings concerning the specificity and privilege of the *Cox1* gene sequences for the *Sarcocystis* species characterization were concordant with the previous investigations (Gjerde et al. 2020; El-Morsey et al. 2021).

The present study fully described and identified for the first time the sarcocysts of *S. hircicanis*, and *S. moulei* from domestic goats (*Capra hiricus*) in Egypt. A comprehensive morphologic and molecular characterization of *S. moulei, S. hircicanis,* and *S. capracanis* on the levels of *18S rRNA, 28S rRNA,* and *Cox1* genes was performed for the first time as well. *Sarcocystis moulei* macrosarcocysts were observed for the first time in Egypt in the cardiac, esophageal, skeletal, diaphragmatic, and lingual muscles of domestic goats (*Capra hiricus*).

### Supplementary information

ESM 1 (DOCX 6496 kb)

## Data Availability

Data supporting the conclusions of the current investigation are included in the article. The sequences generated in this study represent all the isolates of *Sarcocystis* spp. detected herein were submitted to GenBank under the Accession numbers mentioned in Table 4. All the sequences corresponding to the *Sarcocystis* spp. identified here can be accessed by surfing the GenBank Blast link. https://blast.ncbi.nlm.nih.gov/Blast.cgi.
