# Supplementary material for: Recent insights into the morphology, molecular characterization and tissue localization of the caprine Sarcocystis species infecting domestic goats (Capra hiricus): Sarcocystis moulei, Sarcocystis capracanis, and Sarcocystis hircicanis
Source: Parasitol Res. 2023 Dec 16;123(1):55. doi: 10.1007/s00436-023-08063-3 (PMC10724331; doi:10.1007/s00436-023-08063-3)
Supplement: Supplementary file 1 — (DOCX 6496 kb) [file 436_2023_8063_MOESM1_ESM.docx]

**Parasitology Research - The Supplementary Data**

**Recent insights into the morphology, molecular characterization and tissue localization of the caprine *Sarcocystis* species infecting domestic goats (*Capra* *hiricus*); *Sarcocystis moulei*, *Sarcocystis* *capracanis*, and *Sarcocystis* *hircicanis***

**Ahmed El-Morsey*^1^. Walied Abdo^2^**

**^1^**Parasitology and Animal Diseases Department, Veterinary Research Institute, National Research Centre, 33 El Buhouth St. (former El-Tahrir St.), Dokki, P.O. 12622, Giza, Egypt

**^2^**Department of Pathology and Clinical Pathology, Faculty of Veterinary Medicine, KafrElsheikh University, 33516 Kafr El-Sheikh, Egypt

**Corresponding Author,**

**Ahmed El-Morsey***

**Parasitology and Animal Diseases Department, Veterinary Research Institute, National Research Centre, 33 El Buhouth St. (former El-Tahrir St.), Dokki, P.O. 12622, Giza, Egypt**

**Tel: +20233371362; Fax: +20233370931C**

[el.el-sayed@nrc.sci.eg](mailto:el.el-sayed@nrc.sci.eg)

[Ahmed El-Morsey (0000-0001-5105-9465) - ORCID](https://orcid.org/0000-0001-5105-9465)

Affiliation City information of the second author; Walied Abdo

Faculty of Veterinary Medicine, Mubarak Rd, Kafr Abu Tabl, Kafr Al Sheikh First, Kafr El Sheikh Governorate 33516, Egypt.

Tel: [+ 002-047-3109590](https://www.google.com/search?q=%D8%B9%D9%86%D9%88%D8%A7%D9%86+%D9%83%D9%84%D9%8A%D8%A9+%D8%A7%D9%84%D8%B7%D8%A8+%D8%A7%D9%84%D8%A8%D9%8A%D8%B7%D8%B1%D9%89+%D8%AC%D8%A7%D9%85%D8%B9%D8%A9+%D9%83%D9%81%D8%B1+%D8%A7%D9%84%D8%B4%D9%8A%D8%AE&oq=%D8%B9%D9%86%D9%88%D8%A7%D9%86+%D9%83%D9%84%D9%8A%D8%A9+%D8%A7%D9%84%D8%B7%D8%A8+%D8%A7%D9%84%D8%A8%D9%8A%D8%B7%D8%B1%D9%8A+%D8%AC%D8%A7%D9%85%D8%B9%D8%A9+&aqs=chrome.7.69i57j0i19i512l3j0i19i22i30l6.33838j0j15&sourceid=chrome&ie=UTF-8); fax: [+ 002-047-3109591](https://www.google.com/search?q=%D8%B9%D9%86%D9%88%D8%A7%D9%86+%D9%83%D9%84%D9%8A%D8%A9+%D8%A7%D9%84%D8%B7%D8%A8+%D8%A7%D9%84%D8%A8%D9%8A%D8%B7%D8%B1%D9%89+%D8%AC%D8%A7%D9%85%D8%B9%D8%A9+%D9%83%D9%81%D8%B1+%D8%A7%D9%84%D8%B4%D9%8A%D8%AE&oq=%D8%B9%D9%86%D9%88%D8%A7%D9%86+%D9%83%D9%84%D9%8A%D8%A9+%D8%A7%D9%84%D8%B7%D8%A8+%D8%A7%D9%84%D8%A8%D9%8A%D8%B7%D8%B1%D9%8A+%D8%AC%D8%A7%D9%85%D8%B9%D8%A9+&aqs=chrome.7.69i57j0i19i512l3j0i19i22i30l6.33838j0j15&sourceid=chrome&ie=UTF-8)

**Table of contents:**

| Figures | Page Number |
| --- | --- |
| Figure 1SF | **3** |
| Figure 2SF | **5** |
| Figure 3SF | **6** |
| Figure 4SF | **8** |
| Figure 5SF | **9** |
| Figure 6SF | **11** |
| Figure 7SF | **13** |
| Figure 8SF | **15** |
| Figure 9SF | **16** |
| Figure 10SF | **17** |
| Figure 11SF | **18** |
| Figure 12SF | **19** |
| Tables |  |
| Table 5 | **20-21** |
| Table 6 | **22** |

**Figure 1SF:**


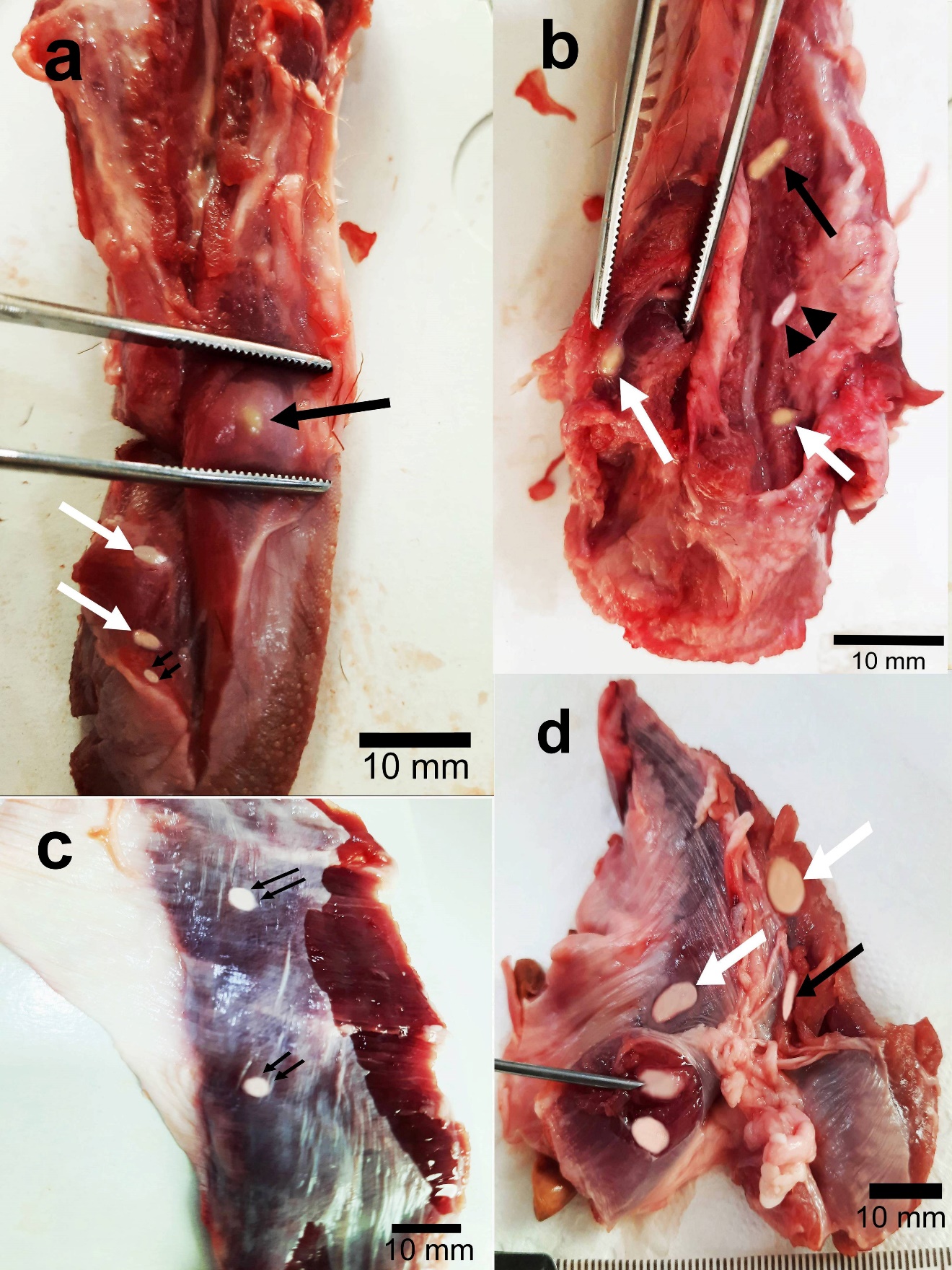


**Fig. 1SF** Macromorphology of *S. moulei* macrosarcocysts in different muscular organs of domestic goats (*Capra hiricus*). (a) Spindle-shaped morphotype (II) of *S. moulei* macrosarcocysts. Whitish sarcocysts are impeded in the interior muscular core of the tongue, (white arrows; double black small arrows). Whereas, yellowish macrocysts were situated on the ventral aspect of the tongue (single black arrow). (b) Yellowish-white ovoid and sub-spherical macrosarcocysts of *S. moulei* located within the musculature of the tongue root (white arrows). Note the white spindle-shaped morphotype (II) sarcocyst located within the muscles of the tongue root (double black arrowheads). Elongated cylindrical yellowish-white morphotype (II) macrocyst of *S. moulei* (black arrow). (c) White oval or spherical macrosarcocysts of *S. moulei*, morphotype (I), located under the serosal sheet of the diaphragm (double black arrows). (d) *Sarcocystis* *moulei* macrocysts are situated in the skeletal muscles of a goat. Large-sized oval or ovoid, spherical yellowish-white or clear white cysts (Morphotype I) (white arrows), and an elongated cylindrical-shaped white cyst of *S. moulei* Morphotype (II) (single black arrow) are observed. Fresh muscular tissues. No stain. Scale bars = 10 mm.

**Figure 2SF:**


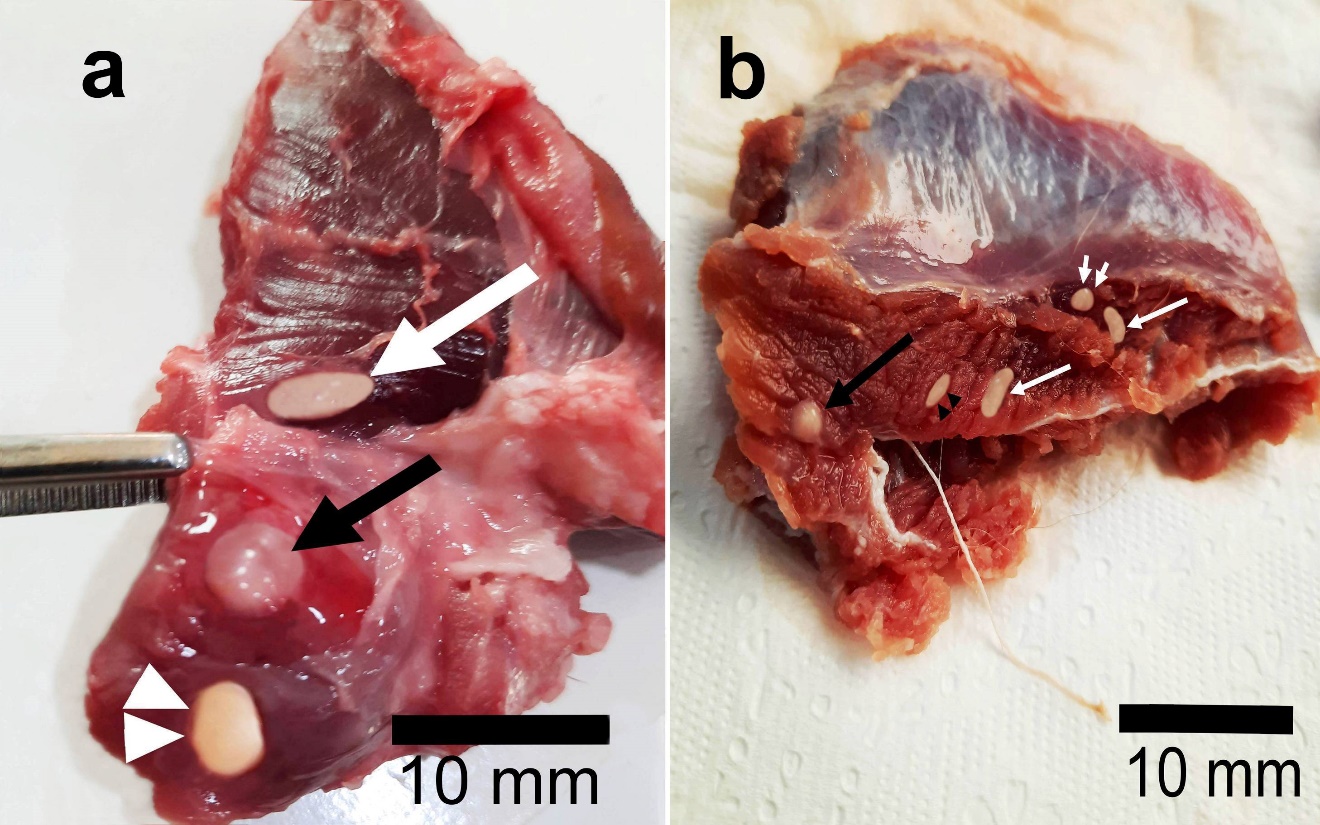


**Fig. 2SF** Gross morphology of *S. moulei* macrosarcocysts in the caprine skeletal muscles. (a) Macrosarcocysts of *S. moulei* (morphotype I) might be yellowish white (two white arrowheads), spherical clear white cysts (black arrow), and opaque white large oval macrocysts (white arrow). (b) Different morphologic forms of *S. moulei* sarcocysts in a skeletal muscle include cylindrical (white single arrows) or spindle-shaped cysts (double black arrowheads) (Morphotype II). Small ovoid and spheroid cysts (double small white arrows and a single black arrow) refer to small-sized early developing morphotypes (I). Fresh skeletal muscle samples. No stain. Scale Bars = 10 mm.

**Figure 3SF:**


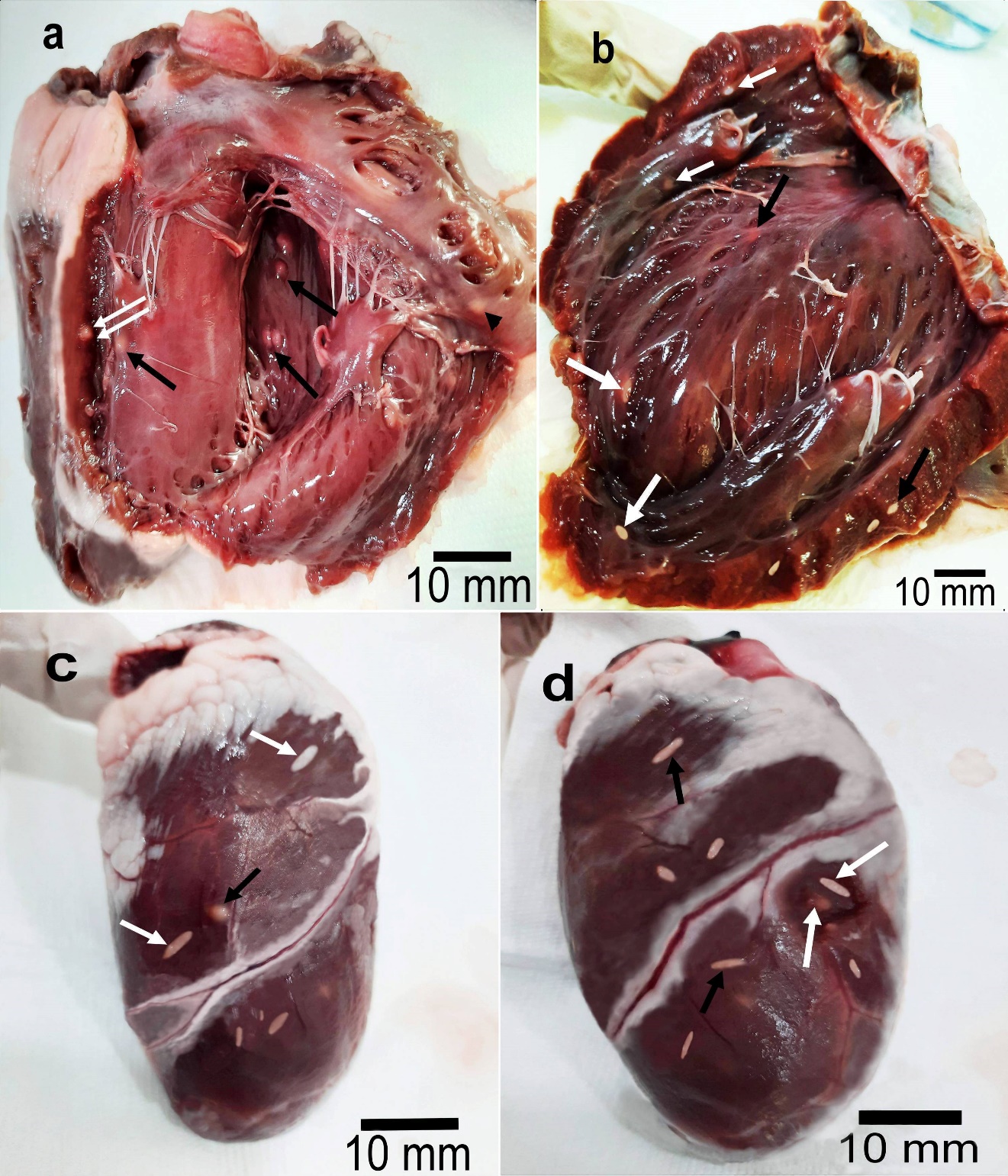


**Fig. 3SF** The macromorphology of *S. moulei* macrosarcocysts (morphotype II) in the caprine cardiac muscles. (a) Spindle shape morphotype (II) of *S. moulei* cysts located under the endocardium of the right ventricle of the heart (single black arrows), small spherical cysts situated within the myocardium (double white arrows), and a spindle-shaped macrosarcocyst in the auricular wall (black arrowhead). (b) Small sized spindle-shaped white cysts of *S. moulei* under the endocardium of the left ventricle of the heart (white arrows) and yellowish-white spindle shape sarcocysts within the myocardium (black arrows). (c) An oval or sub spherical yellowish-white macrosarcocyst of *S. moulei* is seen under the epicardium (may be morphotype I) (black arrow). Elongated spindle-shaped white or yellowish-white macrocysts of *S. moulei* morphotype (II) are seen under the epicardium (White arrows). (d) Elongated spindle-shaped *S. moulei* (morphotype II) cysts located within the myocardium (white arrows). Also, notice the elongated spindle-shaped macrocysts of *S. moulei* morphotype (II) that are seen under the epicardium (Black arrows). Fresh cardiac muscles. No stain. Scale bars 10 = mm.

**Figure 4SF:**


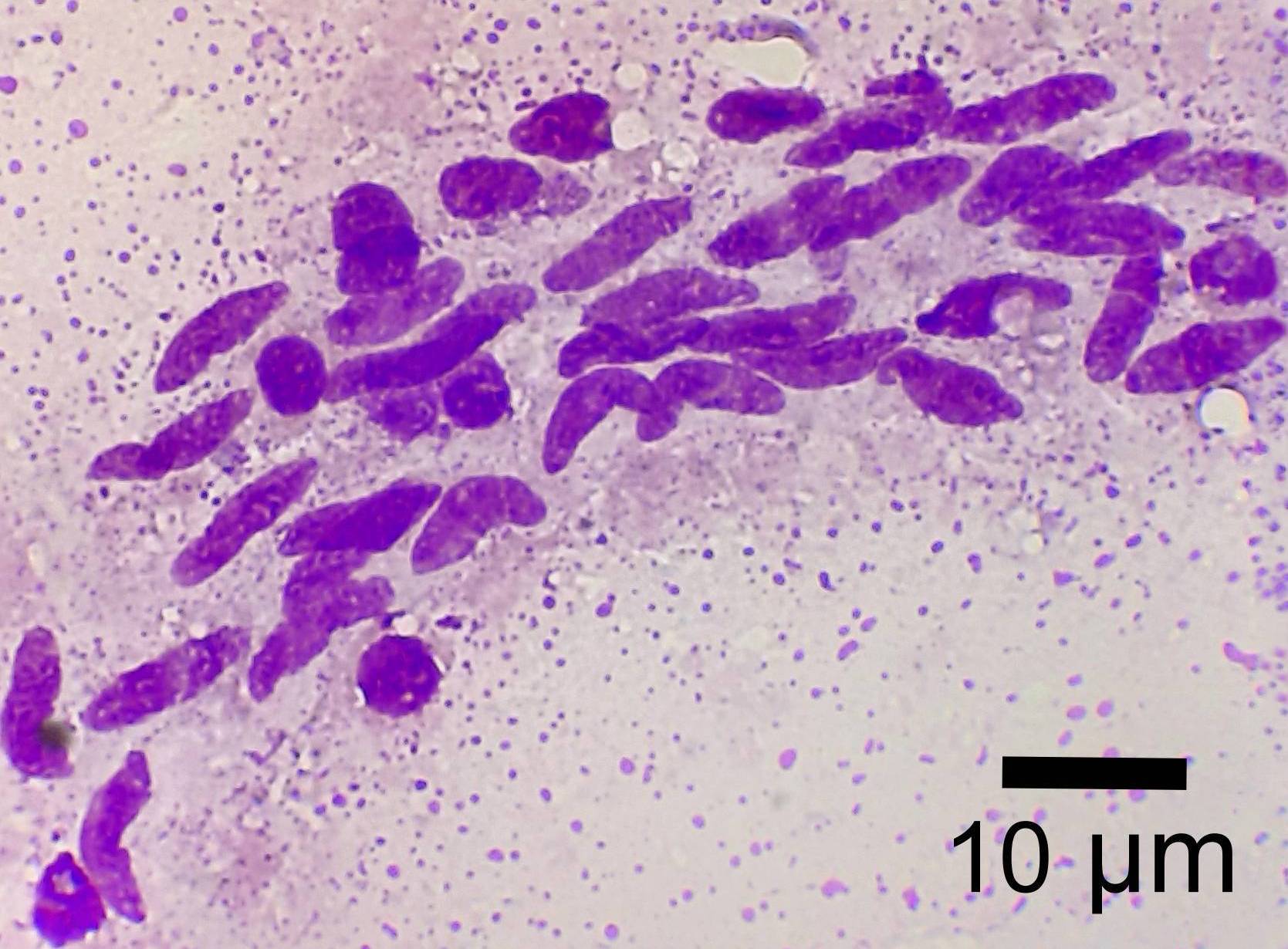


**Fig. 4SF** A microphotograph depicting an impression smear from the heart macrosarcocysts showing banana or crescent-shaped bradyzoites and few oval or globular metrocytes of *S. moulei*. Stain; Giemsa stain. Scale bar = 10 µm.

**Figure 5SF:**


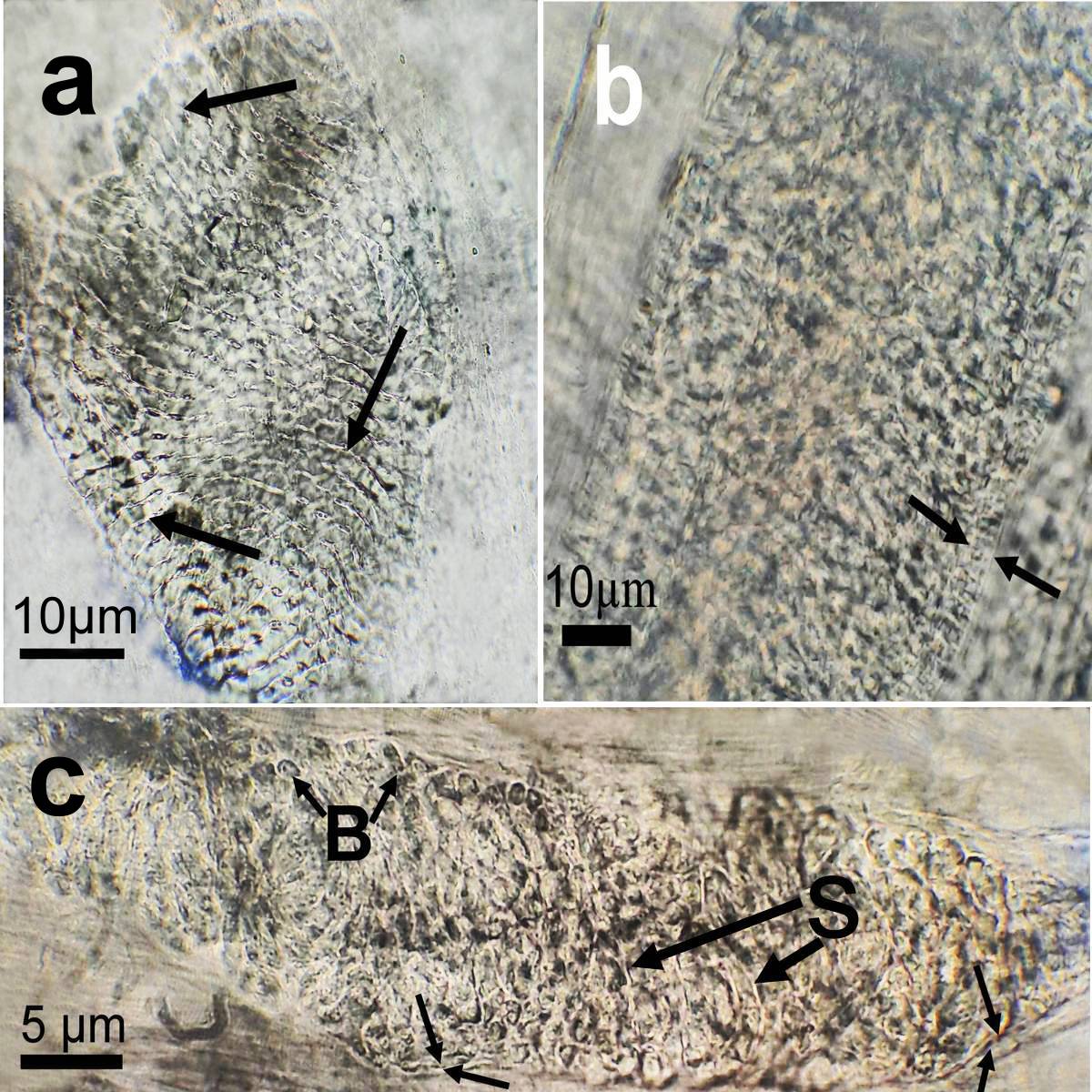


**Fig. 5SF** Photomicrographs depicting the micromorphology of the caprine *Sarcocystis* spp. microcysts in fresh muscle squashes under the light microscope. (a) A compressed *S. capracanis* cyst in an irregular pyriform shape with clearly evident elongated finger-like or cylindrical villar protrusions (VP) on the outer surface of the sarcocyst (black arrows). Scale Bar = 10 µm. (b) A ribbon-shaped cyst of *S. capracanis* in a longitudinal side view with palisade-like VP on the cyst wall (two opposite arrows). Scale bar = 10 µm. (c) *Sarcocystis hircicanis* cyst with a thin smooth wall that has some areas of slight thickening representing the hairy protrusions adhering to the sarcocyst surface (opposing arrows). Bradyzoites (B) and septa (S) are seen from inside the sarcocyst as a consequence of the thin cyst wall. Fresh muscle squashes. No stain. Scale bar = 5 µm.

**Figure 6SF:**


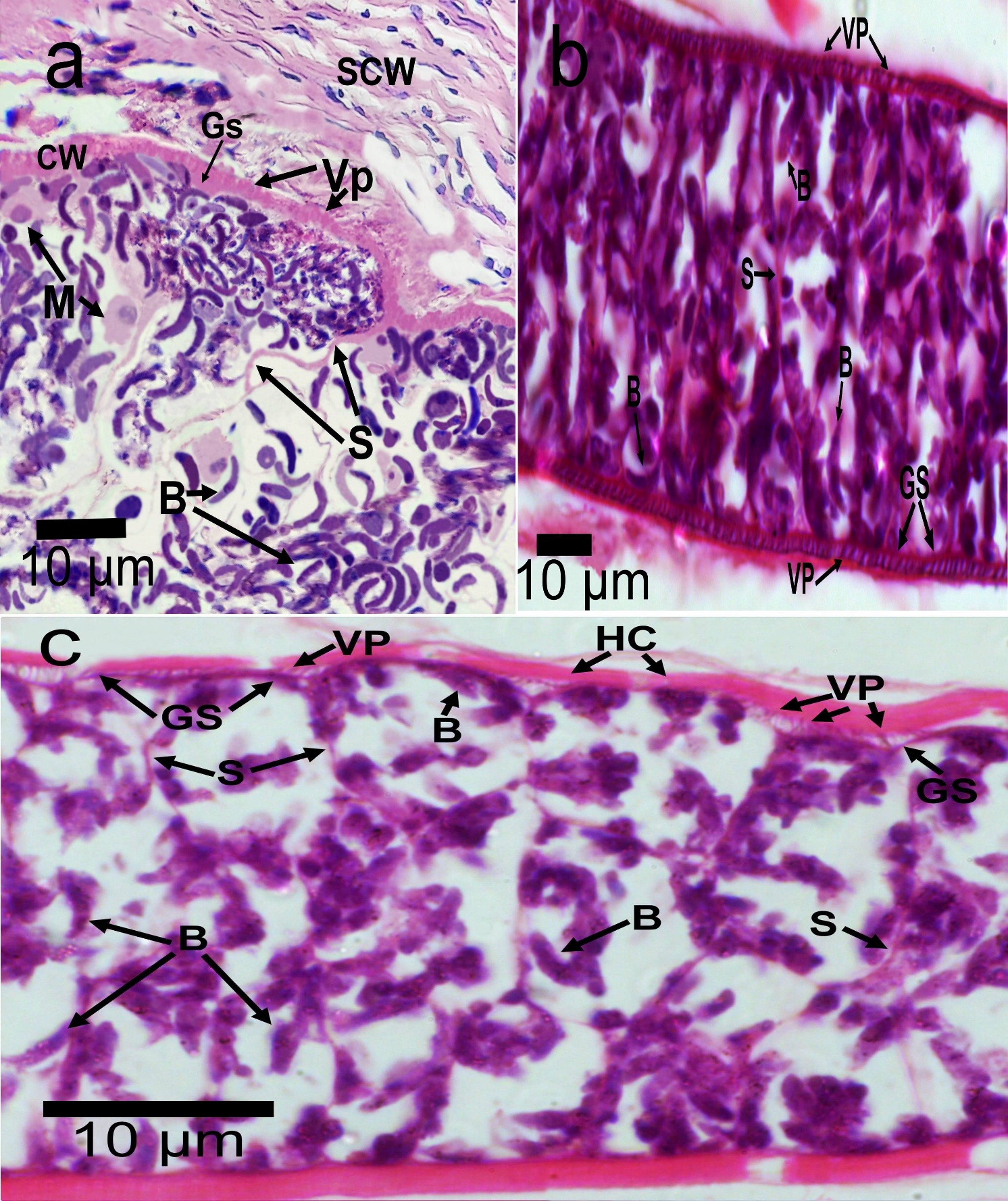


**Fig. 6SF** Micrographs depicting the histopathology of *S. moulei, S. capracanis* and *S. hircicanis* by light microscope. (a) Histologic section of *S. moulei* with corrugated cyst wall (CW) that has small branched VP and outer thick fibrous connective tissue capsule (FCT) formed of collagen fibers and fibrocytes with the existence of degenerated muscle fibers i.e. the secondary cyst wall (SCW). Note the eosinophilic ground substance (GS), the primary sarcocyst wall (CW), well-defined septa (S), and lightly stained (eosinophilic) oval or irregularly shaped metrocytes (M) with centrally located basophilic nuclei, elongated sickle shape bradyzoites (B). Stain H&E. Scale Bar = 10 µm. (b) *Sarcocystis capracanis* cyst with a wall having palisade-like or striated, due to the existence of finger-like protrusions (VP), eosinophilic well-defined ground substance (GS), septa (S), and banana-shaped bradyzoites (B). Stain H&E. Scale Bar = 10 µm. (c) *Sarcocystis hircicanis* cyst that has thin villar protrusions (VP) that get adhered with the cyst wall in some regions giving it a smooth appearance, eosinophilic ground substance (GS), Bradyzoites (B), and septa (S). Stain H&E. Scale Bar = 10 µm.

**Figure 7SF:**

**
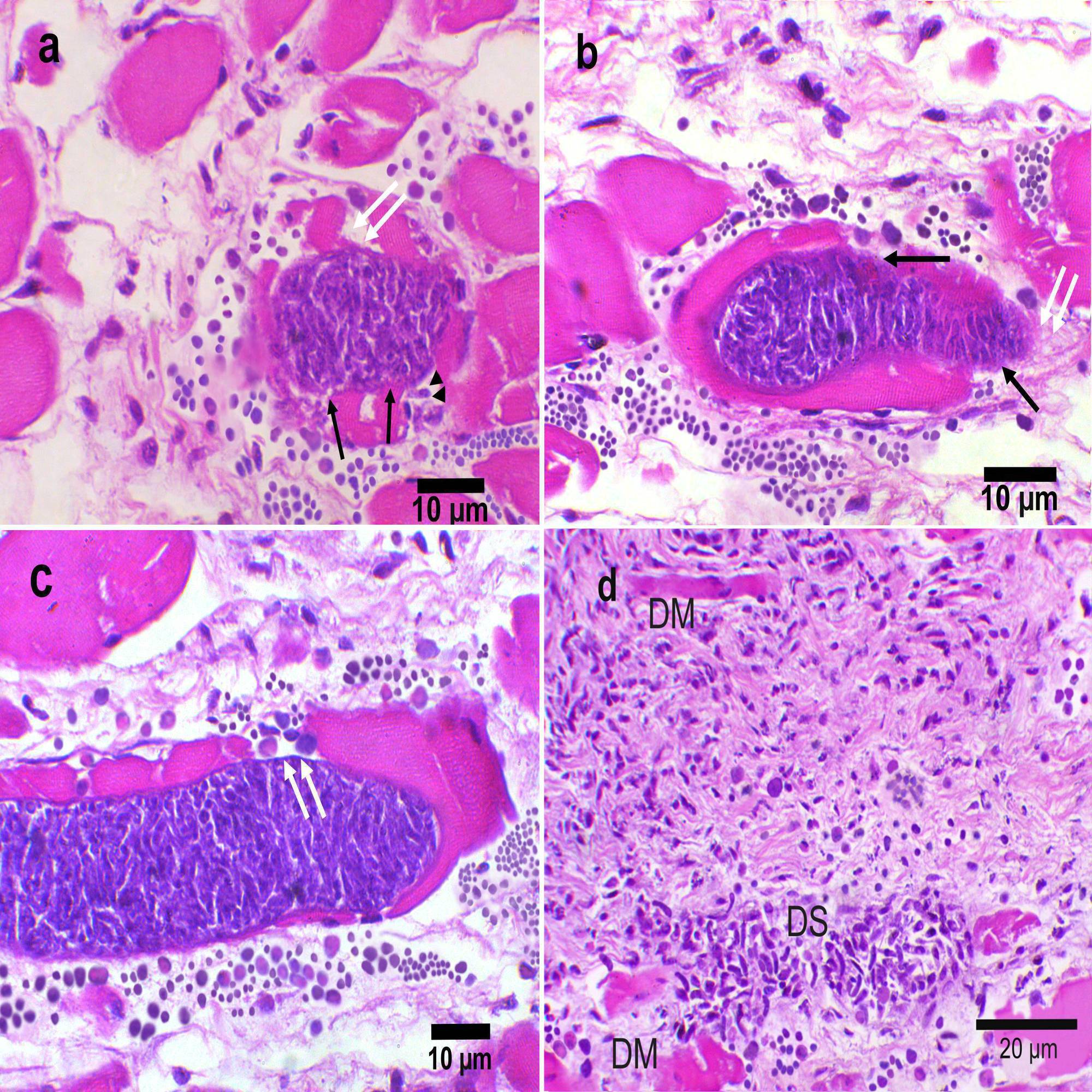
**

**Fig. 7SF** Photomicrographs showing variant degrees of caprine sarcocystosis caused by *S. capracanis* infection in the cardiac muscles of a goat. Figures (a), (b), and (c) depict different three microsarcocysts of *S. capracanis* in the cardiac muscles with variable degrees of degeneration in the sarcocyst walls and migrating or infiltrating mononuclear inflammatory cells as macrophages and lymphocytes in addition to eosinophils attacking the sarcocyst wall. Note degenerated cyst walls in figures (a) and (b) represented by single black arrows. The two black arrowheads refer to a bradyzoite that is getting out from the damaged sarcocyst wall in Fig (a). Double white arrows refer to the degenerating cardiomyocyte enclosing the sarcocyst in figures (a) and (b). Meanwhile, the double white arrows in Figure (c) refer to the onset of thinning or dissociation of the sarcocyst wall together with degeneration of the cardiomyocyte harboring the sarcocyst as a result of the infiltrating macrophages and eosinophils. Degeneration and necrosis of the infected cardiomyocytes harboring the three sarcocysts in addition to complete necrosis of the cardiomyocytes located around the sarcocysts are observed in Figures (a), (b), and (c). Stain H&E. Scale bars = 10 µm. (d) A degenerating sarcocyst (DS) within the cardiac muscles surrounded by completely degenerated and necrosed cardiomyocytes (DM) together with chronic inflammatory cell infiltration that is mainly composed of lymphocytes, macrophages, and a few numbers of eosinophils. Additionally, a high degree of fibrosis of the muscle fibers around the lesion is observed. Stain H&E. Scale bar = 20 µm.

**Figure 8SF:**


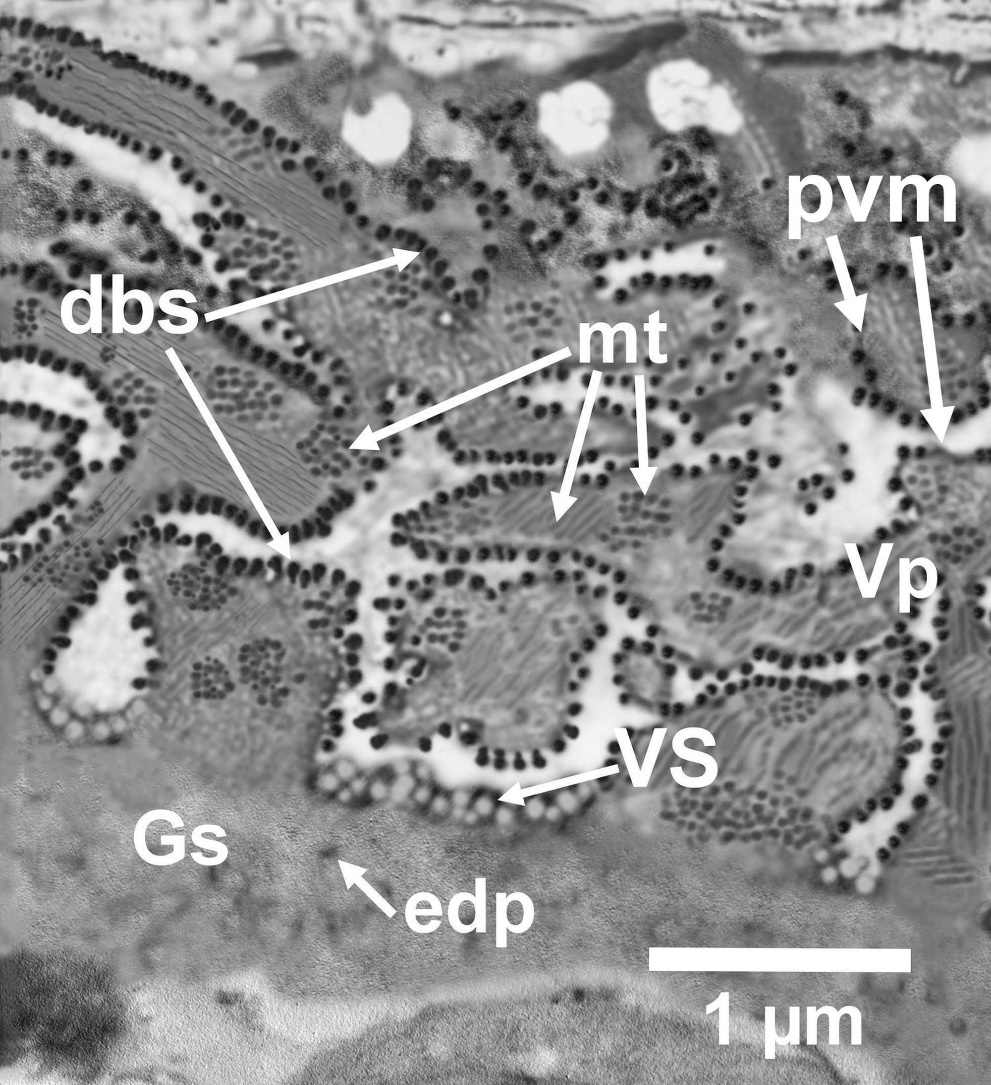


**Fig. 8SF** Higher magnification of *S. moulei* cyst wall with villar protrusion (Vp) that has characteristic dumbbell-like structures (dbs) on the outer surface of the parasitophorous vacuolar membrane (pvm). Note the interior of the (Vp) that is filled with well-developed microtubules in longitudinal and cross arrangements. Notice the two rows of spherical vesicular structures (VS) that are located on the (pvm) in the interspaces between the (Vp), and the electron dense particles (edp) dispersed inside the Ground substance (Gs). Scale bar = 1 µm.

**Figure 9SF:**


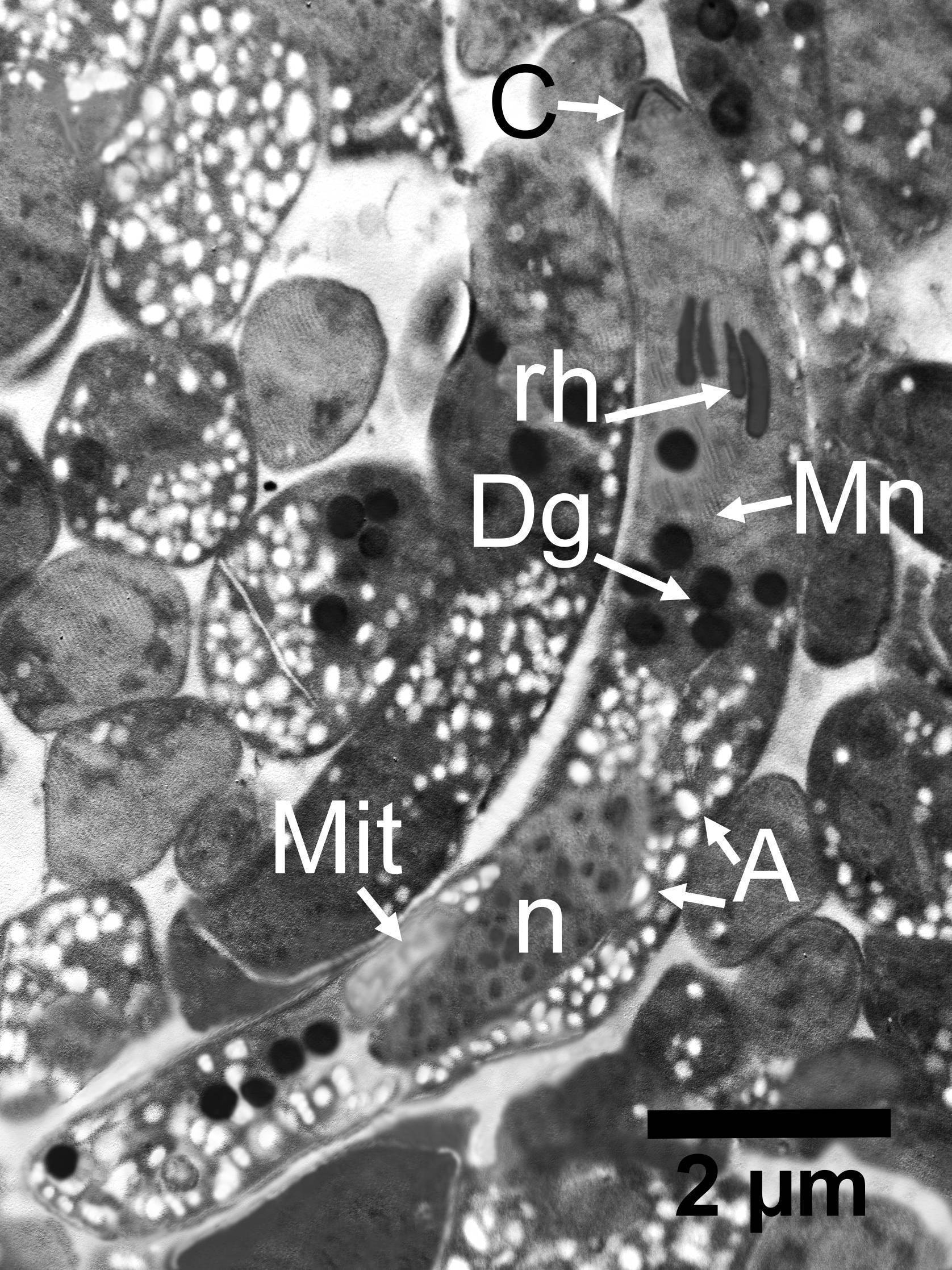


**Fig. 9SF** A micrograph depicting *S. moulei* elongated sickle-shaped bradyzoite with centrally located nucleus (n), anterior conoid (C), 4 rhoptries (rh), small-sized micronemes (Mn), dense granules (Dg), amylopectin granules (A), and mitochondoria (Mit). Scale bar = 2 µm.

**Figure 10SF:**


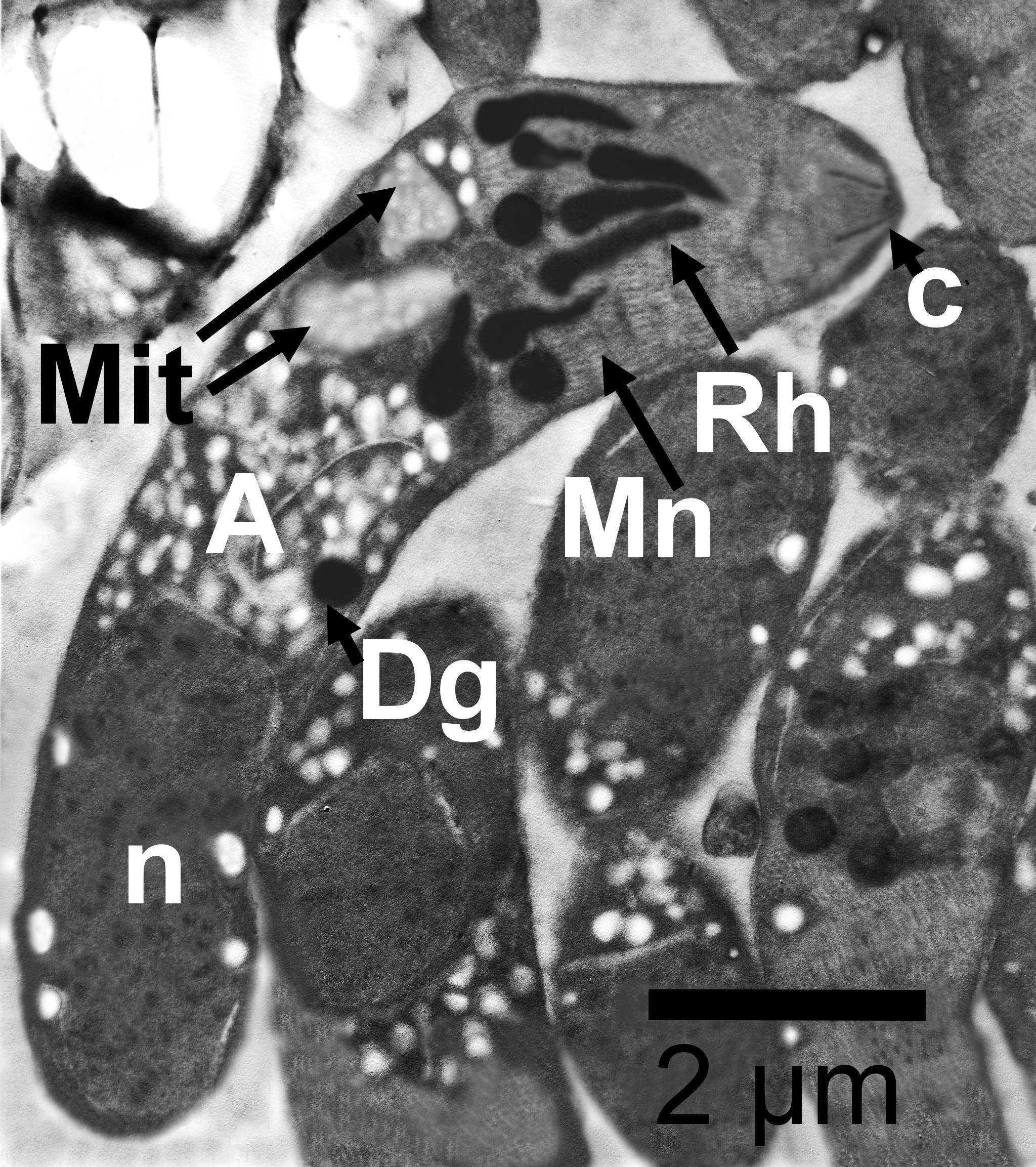


**Fig. 10SF** Banana-shaped bradyzoite of *S. capracanis* that has many organelles of the apical complex comprising here the conoid (c), 7 rhoptries (Rh), micronemes (Mn). In addition, amylopectin granules (A) and mitochondoria (Mit) in front of the posteriorly located nucleus (n) were also observed. Scale bar = 2 µm.

**Figure 11SF:**


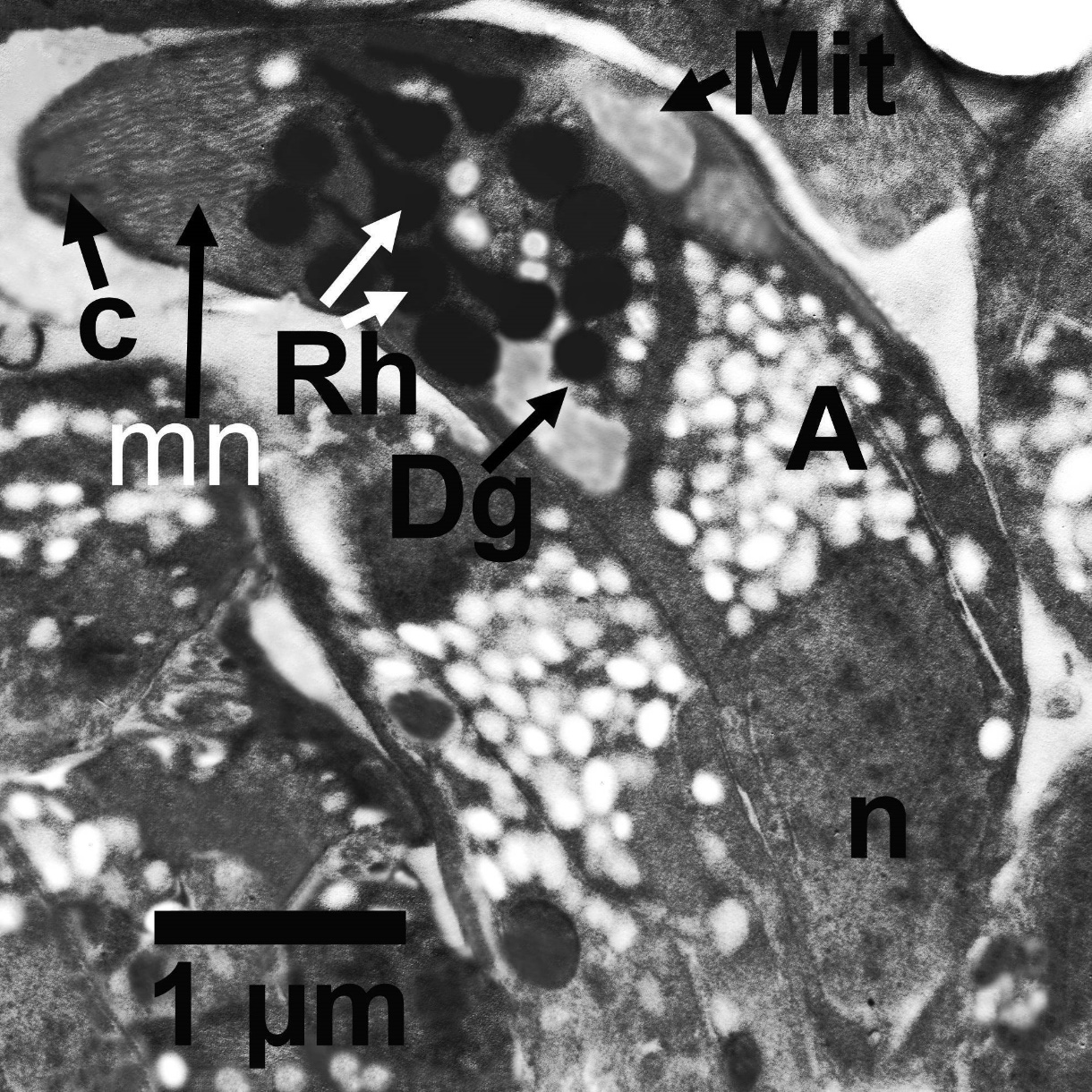


**Fig. 11SF** A photomicrograph depicting a *S. hircicanis* short banana-like bradyzoite with anteriorly located conoid (c), micronemes (mn), 5 drum-stick like rhoptries (Rh), dense granules (Dg), amylopectin granules (A), mitochondoria (Mit) and posterior nucleus (n). Scale bar = 1 µm.

**Figure 12SF:**

. **
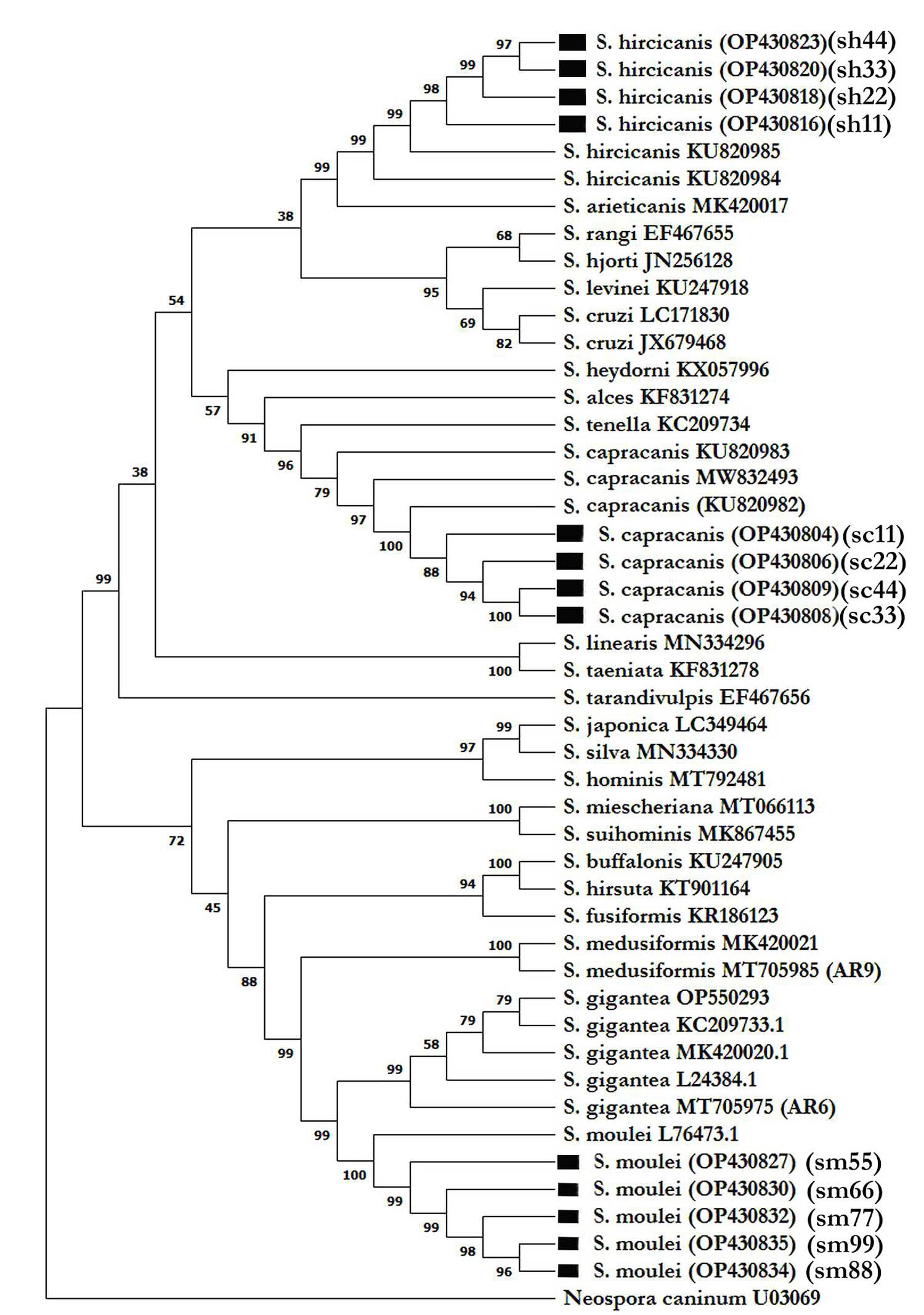
**

**Fig. 12SF** A cladogram for the *18S rRNA* gene sequences of the *Sarcocystis* species of ruminants. Note the robust association of *S. moulei, S. capracanis*, and *S. hircicanis* isolates identified in the present study with the previously detected isolates of the same species. The tree was reconstructed using the minimum evolution (ME) method and rooted on *Neospora caninum* isolate (U03069) as outgroup. The newly detected isolates herein, are marked by black squares immediately after the branch ends just before the taxa names. Whereas, the haplotype names are mentioned in parenthesis next to the GenBank accession numbers

**Table 5** Identities of the 3 *Sarcocystis* spp*. 18S rRNA* and *28S rRNA* isolates identified here compared to the previous sequences. The present isolates accession numbers are in boldface.

|  | *18S rRNA* isolates | | | *28S rRNA* isolates | | |
| --- | --- | --- | --- | --- | --- | --- |
| *S. moulei* | Current | Previous | Identity % | Current | Previous | Identity % |
|  | **OP430827 (sm55)** | *S. moulei*  L76473 | (99.78%) | **OP429586 (SM116)** | *S. moulei* AF012884 | (99.97%) |
|  | **OP430830 (sm66)** |  | (99.89%) | **OP430799 (SM18)** |  | (99.91%) |
|  | **OP430832 (sm77)** |  | (99.62%) | **OP430800 (SM120)** |  | (99.89%) |
|  | **OP430834 (sm88)** |  | (99.46%) | **OP430802 (SM 124)** |  | (99.80%) |
|  | **OP430835 (sm99)** |  | (99.29%) | **OP430801 (SM122)** |  | (99.86%) |
|  |  | *S.gigantea* MK420020 (OS13) | (98.59%) | **OP430803 (SM126)** |  | (99.71%) |
|  |  | *S.gigantea* MT705975 (AR6) | (98.22%) |  | *S.arieticanis=S. moulei* AF044250 | (97.89%) |
|  |  |  |  |  | *S.gigantea* U85706 | (97.89%) |
|  |  |  |  |  | *S.gigantea* MK420025 | (97.87%) |
|  |  |  |  |  | *S.gigantea* MT706045 (AR20) | (97.52%) |
|  |  |  |  |  | *S.medusiformis* MT706454 (AR22) | (94.31%) |
| *S. capracanis* | **OP430804 (sc11)** | *S.capracanis* KU820982 | (99.89%) | **OP425732 (sc100)** | *S.capracanis* KU820978 | (99.91%) |
|  | **OP430806 (sc22)** |  | (99.83%) | **OP425798 (sc102)** |  | (99.91%) |
|  | **OP430808 (sc33)** |  | (99.66%) |  | KU820979 | (99.88%) |
|  |  | *S.capracanis* MW832493 | (99.66%) | **OP425811 (sc104)** |  | (99.74%) |
|  | **OP430809 (sc44)** |  | (99.44%) | **OP426272 (sc106)** |  | 99.45% |
|  |  | *S.capracanis* MW832485 | (99.55%) |  | *S.capracanis* AF012885 | 98.53% |
|  |  | MW832482 | (99.55%) |  | *S.tenella* AF076899 | 98.32% |
|  |  | KU820983 | (99.21)% |  | *S.tenella* MF039326 | 98.32% |
|  |  | L76472 | (99.05)% |  |  |  |
| *S. hircicanis* | **OP430816 (sh11)** | *S.hircicanis* KU820985 | (99.89%) | **OP426444 (sh108)** | *S.hircicanis*  KU820980 | 99.86% |
|  | **OP430818 (sh22)** |  | (99.83%) | **OP429224 (sh110)** |  | 99.86% |
|  | **OP430820 (sh33)** |  | (99.60%) | **OP429423 (sh112)** |  | 99.80% |
|  |  | KU820984 | (99.49%) | **OP429424 ( sh114)** |  | 99.66% |
|  | **OP430823 (sh44)** |  | (99.38%) |  | *S.hircicanis* KU820981 | 98.46% |
|  |  | *S.arieticanis* MK420017 | (98.43%) |  | *S. arieticanis* MF039328 | 95.93% |
|  |  |  |  |  | *S.arieticanis* AF076904 | 95.87% |
|  |  |  |  |  | *S.arieticanis* MH413039 | 94.85% |
|  |  |  |  |  | *S.tenella* AF076899 | 94.44% |
|  |  |  |  |  | *S.capracanis* KU820978 | 94.38% |
|  |  |  |  |  | *S.capracanis* KU820979 | 94.35% |
|  |  |  |  | *S. capracanis*  **OP425732 (sc100)** |  | 94.38% |
|  |  |  |  | *S. capracanis*  **OP425798 (sc102)** |  | 94.35% |

**Table 6** Similarities of *S. moulei, S. capracanis,* and *S. hircicanis* *Cox1* isolates identified herein compared to the previous sequences. The current *Cox1* isolates accession numbers are in boldface.

|  | *Cox1* isolates | | |
| --- | --- | --- | --- |
| *S. moulei* | Current | Previous | Identity % |
|  | **OP485101 (SMn1)** |  | ------------ |
|  | **OP485102 (SMn2)** |  | 99.81% |
|  | **OP485103 (SMn3)** |  | 98.84% |
|  | **OP485105 (SMn4)** |  | 98.75% |
|  | **OP485105 (SMn5)** |  | 98.55% |
|  |  | *S. gigantea*  MK420011 (OS7) | 91.91% |
|  |  | *S. gigantea* MT722969 (AR28) | (91.62%) |
|  |  | *S.gigantea*  MT722970 (AR30) | (91.52%) |
| *S. capracanis* | **OP470343 (SC1)** | *S. capracanis* Isolate 1 China, KU820977 | (99.90%) |
|  | **OP470344 (SC2)** |  | (99.90%) |
|  |  | *S. capracanis* Isolate 2, China, KU820974 | 99.33% |
|  |  | *S.capracanis* isolate (20Ar14.1) MW848337 | (98.83%) |
|  |  | *S. capracanis* Isolate B1 MW768900 | (98.01%) |
|  |  | *S. tenella* (OS105) (MK420005) | (93.65%) |
| *S. hircicanis* | **(OP470341)** **(SH1)** | *S. hircicanis* isolate 2 China, KU820975 | (99.91%) |
|  | **(OP470342) (SH2)** |  | (99.91%) |
|  |  | *S. hircicanis* Isolate 1, China, KU820976 | (98.15%) |
|  |  | *S. arieticanis* (YDSSA1) MF039324 | (92.59%) |
|  |  | *S. arieticanis* (OS41) MK419976 | (92.49%) |
|  |  | *S. arieticanis* (1CSA) MH413047 | (87.60%) |
|  |  | *S. arieticanis* (2CSA) MH413048 | (86.54%) |
